# New Insights on Continuous Renal Replacement Therapy for Acute Respiratory Distress Syndrome: A Systematic Review and Meta‐Analysis

**DOI:** 10.1111/crj.70045

**Published:** 2025-01-02

**Authors:** Siyao Zeng, Shanpeng Cui, Yue Li, Zhipeng Yao, Yunlong Li, Yang Cao, Lianghe Wen, Ming Li, Junbo Zheng, Hongliang Wang

**Affiliations:** ^1^ Graduate School Harbin Medical University Harbin Heilongjiang China; ^2^ Department of Critical Care Medicine Second Affiliated Hospital of Harbin Medical University Harbin Heilongjiang China

**Keywords:** acute respiratory distress syndrome, ARDS, continuous renal replacement therapy, CRRT, meta‐analysis

## Abstract

**Background:**

In recent times, the applications of continuous renal replacement therapy (CRRT) beyond kidney‐related conditions have been progressively increasing, and its implementation in randomized controlled trials (RCTs) specifically for acute respiratory distress syndrome (ARDS) has been documented. This meta‐analysis compiles all existing RCTs to assess whether CRRT benefits ARDS.

**Methods:**

We searched 12 databases in English and Chinese and two clinical trial centers up to November 28, 2023. The main outcome indicator is the mortality rate. Secondary outcome indicators include incidence of ventilator‐associated pneumonia (VAP), ICU length of stay, mechanical ventilation time, oxygenation index (OI) at 24 h (h), OI at 48 h, OI at 72 h, OI at 7 days (d), partial pressure of oxygen (PaO_2_) at 72 h, Acute Physiology and Chronic Health Evaluation II (APACHE II) score at 24 h, APACHE II score at 48 h, APACHE II score at 72 h, APACHE II score at 7 d, extravascular lung water indexes (EVLWI) at 72 h, TNF‐α at 24 h, TNF‐α at 7 d, IL‐6 at 24 h, IL‐6 at 48 h, IL‐6 at 72 h, and IL‐6 at 7 d. Statistical measures utilized include risk ratios (RR), weighted mean difference (WMD), and 95% confidence intervals (95% CI).

**Results:**

We summarized 36 studies, including 2123 patients. It was found that for ARDS, using CRRT in addition to conventional therapy can reduce the mortality rate (*I* ^2^ = 0%; RR: 0.40; 95% CI: 0.30–0.53; *p* < 0.01), the incidence of VAP (*I* ^2^ = 0%; RR: 0.44; 95% CI: 0.33–0.59; *p* < 0.01), ICU length of stay, mechanical ventilation time, and EVLWI at 72 h, as well as APACHE II score, TNF‐α, and IL‐6 at various time points. Additionally, it can increase OI during different time intervals and PaO_2_ at 72 h.

**Conclusions:**

Low‐quality evidence suggests that compared with conventional therapy alone, the use of CRRT may be associated with a lower mortality rate, the incidence of VAP, ICU length of stay, mechanical ventilation time, EVLWI, APACHE II score, TNF‐α, and IL‐6 and may be related to better respiratory function. CRRT may be beneficial for ARDS patients. Future multicenter, well‐designed, high‐quality RCTs are needed to substantiate these findings.

## Graphical abstract

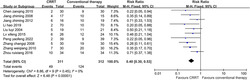

**Abbreviations**
AKI: acute kidney injury
APACHE II: Acute Physiology and Chronic Health Evaluation II
ARDS: acute respiratory distress syndrome
CBM: Chinese Biomedical Literature
CNKI: Chinese National Knowledge Infrastructure
CRRT: continuous renal replacement therapy
CVVH: continuous veno‐venous hemofiltration
CVVHDF: continuous veno‐venous hemodiafiltration
d: days
ESICM: European Society of Intensive Care Medicine
EVLWI: extravascular lung water indexes
FACTT: Fluid and Catheter Treatment Trial
h: hours
ICS: Intensive Care Society
ICU: intensive care units
IL‐6: Interleukin‐6
JRS: Japanese Respiratory Society
MeSH: Medical Subject Headings
MODS: multiple organ dysfunction syndrome
OI: oxygenation index
PaO_2_
: partial pressure of oxygen
PRISMA: Preferred Reporting Items for Systematic Reviews and Meta‐Analyses
RCT: randomized controlled trials
RR: risk ratios
TNF‐α: tumor necrosis factor‐α
VAP: ventilator‐associated pneumonia
VIP: China Science and Technology Journal Database
WMD: weighted mean difference
95% CI: 95% confidence intervals

## 1 Introduction

Acute respiratory distress syndrome (ARDS) is a clinical syndrome defined by acute hypoxemia caused by noncardiogenic pulmonary edema [1, 2]. The incidence and mortality rates of ARDS are both high, with up to 3 million ARDS patients worldwide each year [3]. According to a survey involving nearly 30 000 patients in 495 intensive care units (ICU) across 50 countries on five continents, the incidence of ARDS in ICU accounts for 10.4% of the total ICU admissions, and the in‐hospital mortality rate for ARDS is 40%. Additionally, only about one‐third of patients meeting the diagnostic criteria are recognized by clinical physicians [3].

The common causes of ARDS include both pulmonary and extrapulmonary factors. Pulmonary factors primarily involve pneumonia and chest trauma. Extrapulmonary factors encompass sepsis, pancreatitis, trauma, burns, shock, multiple blood transfusions, aspiration, medications, and toxins [4, 5, 6]. The pathological manifestation of ARDS is characterized by diffuse alveolar damage, where any of the aforementioned clinical injuries can activate alveolar macrophages, neutrophils, and monocytes, releasing pro‐inflammatory cytokines such as tumor necrosis factor‐α (TNF‐α) and interleukin‐6 (IL‐6), causing a “cytokine storm.” These cytokines attract inflammatory cells to the lungs, releasing toxic mediators damaging the endothelium of capillaries and alveolar cells. This results in increased permeability of pulmonary capillaries, leading to the filling of alveoli with proteinaceous fluid. Once alveolar edema surpasses the supportive capacity of surfactant, alveolar cells collapse and undergo necrosis. Additionally, microthrombi may form within the capillaries of the alveoli. These changes ultimately lead to pulmonary edema, impacting gas exchange [1, 4, 7, 8].

Currently, there is no specific therapy for ARDS [4, 6]. Mechanical ventilation is the cornerstone of ARDS treatment. Additionally, treating the underlying condition that leads to ARDS is crucial [9, 10]. Despite advancements in the methods for treating ARDS, which have significantly improved outcomes, the mortality rate remains very high [6].

ARDS often accompanies injuries to other organs, such as acute kidney injury (AKI) [6]. Research indicates that up to 35% of ARDS patients may experience AKI during their time in the ICU [11]. Continuous renal replacement therapy (CRRT) is a frequently used treatment approach for AKI patients in the ICU. In recent years, CRRT has also been extended to nonrenal indications, including sepsis, toxicity, burns, trauma, ARDS, and multiple organ dysfunction syndrome (MODS), among other conditions [12, 13, 14]. In theory, CRRT can benefit ARDS patients by continuously removing low to medium molecular weight inflammatory mediators to counteract the “cytokine storm,” eliminating excess fluid in the lungs to alleviate fluid overload, and improving metabolic processes [8, 15, 16]. Although CRRT has not yet been incorporated into the conventional treatment for ARDS, multiple studies suggest that CRRT can improve respiratory function or prognosis in ARDS patients with various etiologies [14, 17, 18, 19, 20, 21]. There are some randomized controlled trials (RCTs) and retrospective studies on CRRT for treating ARDS. The effectiveness of CRRT for ARDS is not yet clear. This meta‐analysis compiles all existing RCTs to assess whether CRRT benefits ARDS.

## 2 Methods

We have registered this meta‐analysis in the PROSPERO registry with the ID CRD42023485928 and carried it out following the Preferred Reporting Items for Systematic Reviews and Meta‐Analyses (PRISMA) guidelines.

### 2.1 Search Strategies

Two members of the review team searched the main five Chinese and seven English databases from inception to November 28, 2023, namely, Chinese National Knowledge Infrastructure (CNKI), Chinese Biomedical Literature (CBM), China Science and Technology Journal Database (VIP), WanFang, Duxiu, Web of Science, Scopus, PubMed, Cochrane, Ovid, ProQuest, and Embase. Furthermore, we searched ClinicalTrials.gov and the Chinese Clinical Trial Registry to locate pertinent clinical trials. The literature search imposes no restrictions on language. In case of any discrepancies encountered during the search process, a third member of the review team will be engaged to address and resolve them. Our literature searches were conducted using Medical Subject Headings (MeSH) terms. The main MeSH terms employed were “ respiratory distress syndrome” and “ continuous renal replacement therapy.” The free terms include “acute respiratory distress syndrome,” “ARDS,” and “continuous blood purification,”. Please refer to Data S1 for an exhaustive search strategy.

### 2.2 Inclusion Criteria

The inclusion criteria are as follows: (1) RCTs; (2) the patient has been diagnosed with ARDS, with or without comorbid AKI. For ARDS, the recent literature adopts the Berlin definition as the diagnostic criteria; older literature may refer to the standards set in the 1994 American–European Consensus Conference [22, 23]; (3) in each study, the control group was given conventional therapy, while the experimental group received CRRT treatment on the basis of conventional treatment.

### 2.3 Exclusion Criteria

The exclusion criteria are as follows: (1) inconsistent disease types; (2) discrepancies in intervention or control measures in each study; (3) nonmatching outcome indicators; (4) the articles comprise reviews or meta‐analyses; (5) inclusion of animal experiments; (6) the study populations in the articles do not consist of adults; (7) unavailability of articles; (8) data duplication in the articles; (9) no data available in the articles; (10) articles are not RCTs.

### 2.4 Definition of Outcome Indicators

The main outcome indicator is the mortality rate. Mortality rate refers to the mortality rate of patients during hospitalization. Secondary outcome indicators include incidence of ventilator‐associated pneumonia (VAP), ICU length of stay, mechanical ventilation time, oxygenation index (OI) at 24 h (h), OI at 48 h, OI at 72 h, OI at 7 days (d), partial pressure of oxygen (PaO_2_) at 72 h, Acute Physiology and Chronic Health Evaluation II (APACHE II) score at 24 h, APACHE II score at 48 h, APACHE II score at 72 h, APACHE II score at 7 d, extravascular lung water indexes (EVLWI) at 72 h, TNF‐α at 24 h, TNF‐α at 7 d, IL‐6 at 24 h, IL‐6 at 48 h, IL‐6 at 72 h, and IL‐6 at 7 d. The outcome indicators at these respective time points are obtained after treatment. We compare the indicators at these time points between the experimental and control groups to assess the efficacy of the treatments.

### 2.5 Literature Screening and Data Extraction

Two reviewers worked together to screen articles and extract data, with the additional task of conducting checks assigned to the other two reviewers. In the event of any issues, the fifth reviewer would be involved to assist in their resolution. A table was formulated to record the subsequent data from each study, including primary author and publication year, location, sample size, gender distribution, mean age, etiology of ARDS, and study design. Another table documents CRRT modes, blood flow, replacement fluid flow, ultrafiltration rate, treatment time, treatment frequency, and conventional therapy methods.

### 2.6 Quality Assessment

Two reviewers independently applied the Cochrane Collaboration tool to assess the quality of the studies. If discrepancies arise, two additional reviewers will participate in the discussion to reach a consensus.

### 2.7 Statistical Analysis

The analysis utilized RevMan software (version 5.3; The Nordic Cochrane Center, the Cochrane Collaboration, Copenhagen, Denmark) and Stata software (versions 14 and 17; StataCorp, College Station, TX, USA). Risk ratios (RR) with 95% confidence intervals (95% CI) were calculated for binary variables, and the weighted mean difference (WMD) with 95% CI was used for continuous variables. Heterogeneity was evaluated through the *I* ^2^ test. When substantial heterogeneity was detected among studies (*I* ^2^ > 50%, *p* < 0.05), a random‐effects model was applied to derive the final effect sizes. A fixed‐effects model was adopted in cases of little or no heterogeneity. Subgroup analysis of mortality was conducted based on the duration of CRRT. Egger's test was conducted on outcome indicators from more than three studies to check for publication bias. If publication bias was confirmed (Egger's test *p* value < 0.05), the trim‐and‐fill method was implemented to correct or lessen its impact. Sensitivity analysis was carried out to examine the robustness of the results by sequentially omitting each study involved in the outcome indicators. Studies lacking data were excluded to avoid reporting biases.

## 3 Results

### 3.1 Literature Retrieval

**FIGURE 1:**
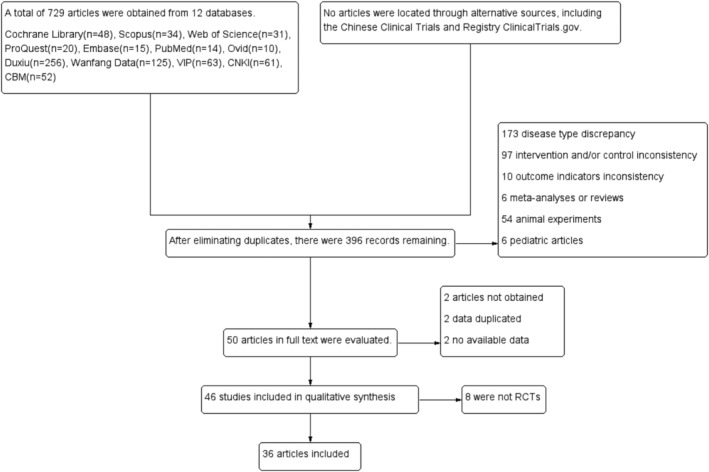
Literature screening flow chart.

At the outset, 729 studies were initially retrieved from 12 databases, with no studies acquired from the two clinical trial centers. After eliminating duplicate studies, 396 studies were left. Among these, 173 studies exhibited inconsistent disease types, 97 showed intervention or control measures variations, and 10 did not align with the specified outcome indicators. Additionally, 54 studies were related to animal experiments, 6 focused on pediatrics, and 6 were meta‐analyses or reviews. After excluding these selected studies, 50 studies were retained. Furthermore, two studies were inaccessible, data from two studies were duplicated, and two studies had no usable data. After excluding these, 46 studies remained. Ultimately, 8 studies that failed to meet the criteria for RCTs were omitted, leaving 36 studies to be incorporated into the meta‐analysis [20, 24, 25, 26, 27, 28, 29, 30, 31, 32, 33, 34, 35, 36, 37, 38, 39, 40, 41, 42, 43, 44, 45, 46, 47, 48, 49, 50, 51, 52, 53, 54, 55, 56, 57, 58]. Figure 1 depicts the review process.

### 3.2 Characteristics of Included Studies

Our investigation involved the analysis of 2123 patients from 36 studies conducted between 2004 and 2023, with 1066 allocated to the experimental group and 1057 to the control group. Conventional therapy for ARDS was administered to the experimental and control groups, including treatment for the underlying primary disease, mechanical ventilation, fluid management, and maintenance of electrolyte and acid–base balance. In addition to these conventional treatments, the experimental group also underwent CRRT. Among the 36 studies included, 4 studies explicitly excluded cases with concomitant AKI, whereas the remaining 32 studies did not specify whether ARDS patients had concurrent AKI. Additionally, 23 studies utilized the continuous veno‐venous hemofiltration (CVVH) mode, 2 studies employed the continuous veno‐venous hemodiafiltration (CVVHDF) mode, 1 study utilized either CVVH or CVVHDF mode, and the remaining 10 studies did not specify the CRRT mode used. There were no notable disparities in the baseline data between the experimental and control groups in each study (*p* > 0.05). The baseline information of the studies included is shown in Table 1 while Table 2 presents the intervention and control measures of each study.

**TABLE 1:** Basic information of each study.

Number	Study and year	Location	Sample size (*N*)	Gender distribution	Mean age (*x* ± *s* or range, years)	Etiology						Study design
						Sepsis	Acute pancreatitis	Pneumonia	Trauma	Burn	Others	
NO.1	Peng Yaofeng 2022	China	*N* = 78 (E: 39; C: 39)	E:M/F: 18/21 C:M/F: 17/22	E: (47.0 ± 3.2) C: (47.4 ± 3.8)	E:7 C:8	E:10 C:11	E:22 C:20	NA	NA	NA	Single center
NO.2	Zheng Wei 2022	China	*N* = 30 (E: 15; C: 15)	E:M/F: 8/7 C:M/F: 7/8	E: (38.4 ± 3.4) C: (38.1 ± 3.6)	E:15 C:15	NA	NA	NA	NA	NA	Single center
NO.3	Hu Manlin 2021	China	*N* = 80 (E: 40; C: 40)	E:M/F: 26/14 C:M/F: 22/18	E: (55.2 ± 17.7) C: (53.2 ± 18.3)	NA	NA	NA	NA	NA	NA	Single center
NO.4	Li Qiuhong 2021	China	*N* = 86 (E: 43; C: 43)	E:M/F: 28/15 C:M/F: 27/16	E: (46.7 ± 5.2) C: (45.8 ± 4.8)	E:43 C:43	NA	NA	NA	NA	NA	Single center
NO.5	Yi Yahui 2021	China	*N* = 112 (E: 56; C: 56)	E:M/F: 30/26 C:M/F: 29/27	E: (50 ± 18) C: (53 ± 18)	NA	NA	NA	E:56 C:56	NA	NA	Single center
NO.6	Yu Jiannan 2021	China	*N* = 75 (E: 36; C: 39)	E:M/F: 19/17 C:M/F: 20/19	E: (20–62) C: (18–60)	E:36 C:39	NA	NA	NA	NA	NA	Single center
NO.7	Li Guohui 2020	China	*N* = 90 (E: 45; C: 45)	E:M/F: 24/21 C:M/F: 23/22	E: (49.7 ± 4.2) C: (48.6 ± 4.3)	NA	NA	NA	NA	NA	NA	Single center
NO.8	Li Zhenxiao 2020	China	*N* = 89 (E: 46; C: 43)	NA	E: (52.6 ± 6.3) C: (48.2 ± 5.4)	36	8	28	13	NA	4	Single center
NO.9	Li Hao 2019	China	*N* = 140 (E: 70; C: 70)	E:M/F: 44/26 C:M/F: 45/25	E: (45–67) C: (46–69)	NA	NA	NA	NA	NA	NA	Single center
NO.10	Zhang Yan 2019	China	*N* = 82 (E: 41; C: 41)	E: M/F: 26/15 C: M/F: 27/14	E: (46.9 ± 3.7) C: (47.8 ± 4.2)	E:41 C:41	NA	NA	NA	NA	NA	Single center
NO.11	Yue Yun 2018	China	*N* = 64 (E: 32; C: 32)	E: M/F: 21/11 C: M/F: 20/12	E: (37.6 ± 3.8) C: (38.1 ± 3.5)	NA	E:9 C:8	E:6 C:5	E:12 C:11	NA	E:5 C:8	Single center
NO.12	Hou Changquan 2017	China	*N* = 76 (E: 40; C: 36)	E: M/F: 28/12 C: M/F: 25/11	E: (48.9 ± 9.2) C: (47.3 ± 8.5)	NA	NA	NA	NA	NA	NA	Single center
NO.13	Wu Xiandan 2017	China	*N* = 48 (E: 24; C: 24)	E: M/F: 13/11 C: M/F: 14/10	E: (52.7 ± 10.1) C: (50.1 ± 9.9)	NA	E:24 C:24	NA	NA	NA	NA	Single center
NO.14	Meng J.B. 2016	China	*N* = 51 (E: 24; C:27)	E: M/F: 16/8 C: M/F: 17/10	E: (62.8 ± 16.4) C: (58.6 ± 17.8)	E:24 C:27	NA	NA	NA	NA	NA	Single center
NO.15	Li Yi 2016	China	*N* = 50 (E: 25; C: 25)	E: M/F: 14/11 C: M/F: 15/10	E: (57 ± 18) C: (56 ± 17)	E:2 C:3	NA	E:8 C:7	E:12 C:11	NA	E:3 C:4	Single center
NO.16	Zhang Lu 2016	China	*N* = 55 (E: 29; C: 26)	E: M/F: 17/9 C: M/F: 14/12	E: (56.4 ± 12.2) C: (53.1 ± 15.8)	NA	E:29 C:26	NA	NA	NA	NA	Single center
NO.17	Zhao Huijuan 2016	China	*N* = 24 (E: 12; C: 12)	NA	NA	NA	NA	NA	NA	NA	NA	Single center
NO.18	Zheng Weigang 2016	China	*N* = 20 (E: 10; C: 10)	M/F: 8/12	(44.2 ± 10.3)	4	NA	12	3	NA	1	Single center
NO.19	Zhou Ruixiang 2016	China	*N* = 68 (E: 34; C: 34)	E: M/F: 17/17 C: M/F: 19/15	E: (54 ± 14) C: (53 ± 14)	NA	E:10 C:9	E:10 C:10	NA	E:5 C:5	E:9 C:10	Single center
NO.20	Chen Zanqing 2015	China	*N* = 60 (E: 30; C: 30)	E: M/F: 17/13 C: M/F: 18/12	E: (54.3 ± 8.1) C: (54.9 ± 7.9)	NA	E:30 C:30	NA	NA	NA	NA	Single center
NO.21	He Yun (1) 2015	China	N = 80 (E: 40; C: 40)	M/F: 62/18	(49.6 ± 6.7)	E:40 C:40	NA	NA	NA	NA	NA	Single center
NO.22	He Yun (2) 2015	China	N = 24 (E: 12; C: 12)	M/F: 18/6	(34–57)	NA	E:12 C:12	NA	NA	NA	NA	Single center
NO.23	Lv Xifeng 2015	China	N = 50 (E: 26; C: 24)	M/F: 33/17	(45.3 ± 12.3)	E:26 C:24	NA	NA	NA	NA	NA	Single center
NO.24	Wang Feng 2015	China	*N* = 40 (E: 20; C: 20)	E: M/F: 13/7 C: M/F: 14/6	E: (43.2 ± 6.8) C: (42.5 ± 6.6)	NA	NA	NA	NA	E:20 C:20	NA	Single center
NO.25	Wang Huamin 2015	China	*N* = 42 (E: 21; C: 21)	E: M/F: 11/10 C: M/F: 13/8	E: (60.1 ± 22.2) C: (57.6 ± 21.2)	NA	5	16	17	NA	4	Single center
NO.26	Liu Ying 2014	China	*N* = 62 (E: 31; C: 31)	E: M/F: 20/11 C: M/F: 19/12	E: (41.2 ± 9.2) C: (40.8 ± 8.9)	NA	NA	NA	NA	NA	NA	Single center
NO.27	Xie Fengchun 2013	China	N = 42 (E: 21; C: 21)	M/F: 30/12	(27–53)	NA	8	20	1	NA	13	Single center
NO.28	Dai Jingcun 2012	China	*N* = 46 (E: 26; C: 20)	E: M/F: 14/12 C: M/F: 11/9	E: (45 ± 6) C: (46 ± 6)	E:9 C:7	E:4 C:4	NA	E:5 C:5	NA	E:8 C:4	Single center
NO.29	Hong Yong 2012	China	*N* = 56 (E: 28; C: 28)	M/F: 35/21	(49.5 ± 20.3)	NA	E:28 C:28	NA	NA	NA	NA	Single center
NO.30	Jiang Zhiming 2012	China	*N* = 35 (E: 16; C: 19)	E: M/F: 9/7 C:M/F: 10/9	E: (48 ± 11) C: (43 ± 13)	NA	NA	NA	NA	NA	NA	Single center
NO.31	Huang Fang 2010	China	*N* = 36 (E: 18; C: 18)	E: M/F: 13/5 C: M/F: 12/6	E: (44.5 ± 10.0) C: (45.0 ± 12.0)	8	4	12	10	NA	2	Single center
NO.32	Zhang Weiqiang 2010	China	N = 60 (E: 30; C: 30)	M/F: 34/26	(32–76)	12	14	9	15	2	8	Single center
NO.33	Jiang Zhiming 2008	China	*N* = 32 (E: 17; C: 15)	E: M/F: 10/7 C: M/F: 6/9	E: (51.9 ± 12.5) C: (52.4 ± 13.8)	NA	NA	NA	NA	NA	NA	Single center
NO.34	Jin Zhaochen 2008	China	N = 40 (E: 20; C: 20)	E: M/F: 12/8 C: M/F: 14/6	E: (56 ± 17) C: (53 ± 16)	8	9	6	10	1	6	Single center
NO.35	Zhang Changqi 2008	China	N = 60 (E: 30; C: 30)	M/F: 32/28	E: (57.1 ± 18.1) C: (56.3 ± 19.4)	12	11	NA	15	NA	22	Single center
NO.36	Liu Luyi 2004	China	N = 40 (E: 19; C: 21)	M/F: 31/9	(38 ± 12)	NA	NA	NA	NA	NA	NA	Single center

Abbreviations: C, control group; E, experimental group; F, female; NA, not applicable; M, male.

**TABLE 2:** Intervention and control measures of each study.

ID	Study	Intervention					Control
		CRRT					Standard therapy
		Mode	Blood flow rate (mL/min)	Replacement fluid rate	Ultrafiltration rate (mL·kg^−1^·h^−1^)	Treatment time and frequency	
NO.1	Peng Yaofeng 2022	UN	180	1500 (mL/h)	UN	The first treatment was continuous for 24 h, and after stabilization the treatment was 12 h/d for 27 h.	Treatment of primary disease, mechanical ventilation, anti‐infection, maintenance of stable internal environment, and correction of acid–base intoxication.
NO.2	Zheng Wei 2022	CVVHDF	150–200	1500 (mL/h)	38	UN	Treatment of primary disease, mechanical ventilation, fluid management, use of vasoactive drugs, and nutritional support.
NO.3	Hu Manlin 2021	CVVH	180–200	4000 (mL/h)	UN	1 week	Treatment of primary disease, mechanical ventilation, removal of triggers, anti‐infection, anti‐inflammation, nutritional support, and maintenance of electrolyte balance.
NO.4	Li Qiuhong 2021	CVVH	200–250	50–60 (mL/kg/h)	UN	Continuous treatment for more than 24 h.	Treatment of primary disease, lung protective mechanical ventilation, anti‐infection, fluid resuscitation, stabilization of the internal environment, and nutritional support.
NO.5	Yi Yahui 2021	CVVH	150–200	2000–3500 (mL/h)	38	UN	Trauma management, lung protective mechanical ventilation, anti‐infection, supplementation of coagulation factors, temperature management, correction of electrolyte disorders, anti‐shock, and nutritional support.
NO.6	Yu Jiannan 2021	UN	200	3000 (mL/h)	UN	UN	Organ function monitoring and support, etc.
NO.7	Li Quohui 2020	UN	100–200	2000–3500 (mL/h)	UN	48–85 h	Treatment of primary disease, mechanical ventilation, administration of vasoactive drugs, fluid management, and nutritional support.
NO.8	Li Zhenxiao 2020	CVVH	130–180	2500–2800 (mL/h)	UN	Continuous treatment for more than 72 h.	Treatment of primary disease, mechanical ventilation, anti‐infection, fluid management, and nutritional support.
NO.9	Li Hao 2019	UN	UN	UN	UN	48–85 h	Mechanical ventilation and other treatments.
NO.10	Zhang Yan 2019	CVVH	150–200	UN	UN	12 h/d	Treatment of primary disease, mechanical ventilation, anti‐infection, fluid resuscitation, stabilization of the internal environment, and nutritional support.
NO.11	Yue Yun 2018	CVVH	UN	UN	UN	UN	Mechanical ventilation and other treatments.
NO.12	Hou Changquan 2017	CVVH	180–200	UN	UN	20–24 h/d, consecutive for 7 days	Treatment of primary disease, mechanical ventilation, anti‐infection, nutritional support, and correction of acid–base intoxication.
NO.13	Wu Xiandan 2017	UN	180–250	2000–3000 (mL/h)	UN	UN	Treatment of primary disease, mechanical ventilation, nutritional support, anti‐infection, etc.
NO.14	Meng J.B. 2016	CVVH	180–220	35 (mL/kg/min)	UN	UN	Mechanical ventilation, anti‐infection, fluid resuscitation, etc.
NO.15	Li Yi 2016	CVVH	100–150	1000 (mL/h)	UN	UN	Treatment of primary disease, lung protective mechanical ventilation, anti‐infection, fluid management, stabilization of the internal environment, and nutritional support.
NO.16	Zhang Lu 2016	CVVH	180	1000–4000 (mL/h)	UN	Once every 24 h, treat for 12–24 h each time for 3 days.	Treatment of primary disease, mechanical ventilation, nutritional support, anti‐infection, stabilization of the internal environment, etc.
NO.17	Zhao Huijuan 2016	UN	UN	UN	UN	UN	Mechanical ventilation, anti‐infection, fluid management, use of glucocorticoids and diuretics, protection of organ function, and symptomatic supportive treatment.
NO.18	Zheng Weigang 2016	CVVH	220	UN	UN	Perform 2 treatments within 7 days, each lasting 24 h.	Mechanical ventilation, anti‐infection, etc.
NO.19	Zhou Ruixiang 2016	CVVH	150–200	2000–3500 (mL/h)	38	48–85 h	Treatment of primary disease, mechanical ventilation, administration of vasoactive drugs and glucocorticoids, fluid management, and nutritional support.
NO.20	Chen Zanqing 2015	CVVHDF	120–180	UN	UN	Once every 24 h, treat for 12 h each time for 3 days.	Mechanical ventilation, fasting, gastrointestinal decompression, inhibition of pancreatic enzymes, acid suppression, nutritional support, pain relief, anti‐infection, etc.
NO.21	He Yun (1) 2015	UN	250–300	4000 (mL/h)	UN	Continuous treatment for 72 h.	Mechanical ventilation, anti‐infection, fluid resuscitation, nutritional support, maintenance of stable internal environment, and correction of acid–base intoxication.
NO.22	He Yun (2) 2015	CVVH	UN	UN	UN	40–56 h	Treatment of primary disease, mechanical ventilation, anti‐infection, etc.
NO.23	Lv Xifeng 2015	UN	150–280	UN	UN	5–12 d	Treatment of primary disease, mechanical ventilation, anti‐infection, fluid resuscitation, and nutritional support.
NO.24	Wang Feng 2015	CVVH and CVVHDF	UN	UN	UN	8–16 h per day	Mechanical ventilation, anti‐infection, fluid resuscitation, burn wound treatment, nutritional support, organ function protection, antioxidation, glucocorticoids, etc.
NO.25	Wang Huamin 2015	CVVH	150	UN	UN	UN	Treatment of primary disease, mechanical ventilation, anti‐infection, nutritional support, and symptomatic treatment.
NO.26	Liu Ying 2014	UN	200	2500 (mL/h)	UN	8–24 h per day	Treatment of primary disease, mechanical ventilation, maintenance of stable internal environment and correction of acid–base intoxication, etc.
NO.27	Xie Fengchun 2013	CVVH	120–150	1500–2500 (mL/h)	UN	UN	Treatment of primary disease, mechanical ventilation, nutritional support, maintenance of stable internal environment, and correction of acid–base intoxication.
NO.28	Dai Jingcun 2012	CVVH	120–200	1500–3000 (mL/h)	UN	UN	Treatment of primary disease, mechanical ventilation, nutritional support, etc.
NO.29	Hong Yong 2012	UN	250–300	4000 (mL/h)	UN	UN	Treatment of primary disease, mechanical ventilation, nutritional support, anti‐infection, glucocorticoids, etc.
NO.30	Jiang Zhiming 2012	CVVH	120–200	2000–3000 (mL/h)	UN	20 h per day	Mechanical ventilation and other treatments.
NO.31	Huang Fang 2010	CVVH	120–180	35 (mL/kg/min)	UN	UN	Treatment of primary disease, lung protective ventilation, anti‐infection, fluid management, stabilization of the internal environment, and nutritional support.
NO.32	Zhang Weiqiang 2010	CVVH	150–250	UN	UN	Continuous treatment for 72 h.	Treatment of primary disease, mechanical ventilation, anti‐infection, organ function protection, and nutritional support, etc.
NO.33	Jiang Zhiming 2008	CVVH	150–250	UN	UN	Continuous treatment for 72 h.	Mechanical ventilation and other treatments.
NO.34	Jin Zhaochen 2008	CVVH	100–150	1000 (mL/h)	UN	UN	Treatment of primary disease, lung protective ventilation, anti‐infection, fluid management, stabilization of the internal environment, and nutritional support.
NO.35	Zhang Changqi 2008	CVVH	200	3000 (mL/h)	UN	Continuous treatment for 24–72 h.	Mechanical ventilation and other treatments.
NO.36	Liu Luyi 2004	CVVH	200–250	2000–4000 (mL/h)	UN	UN	Mechanical ventilation and other treatments.

Abbreviations: CRRT, continuous renal replacement therapy; CVVH, continuous veno‐venous hemofiltration; CVVHDF, continuous veno‐venous hemodiafiltration; d, days; h, hours; UN, unreported.

### 3.3 Quality Assessment

**FIGURE 2:**
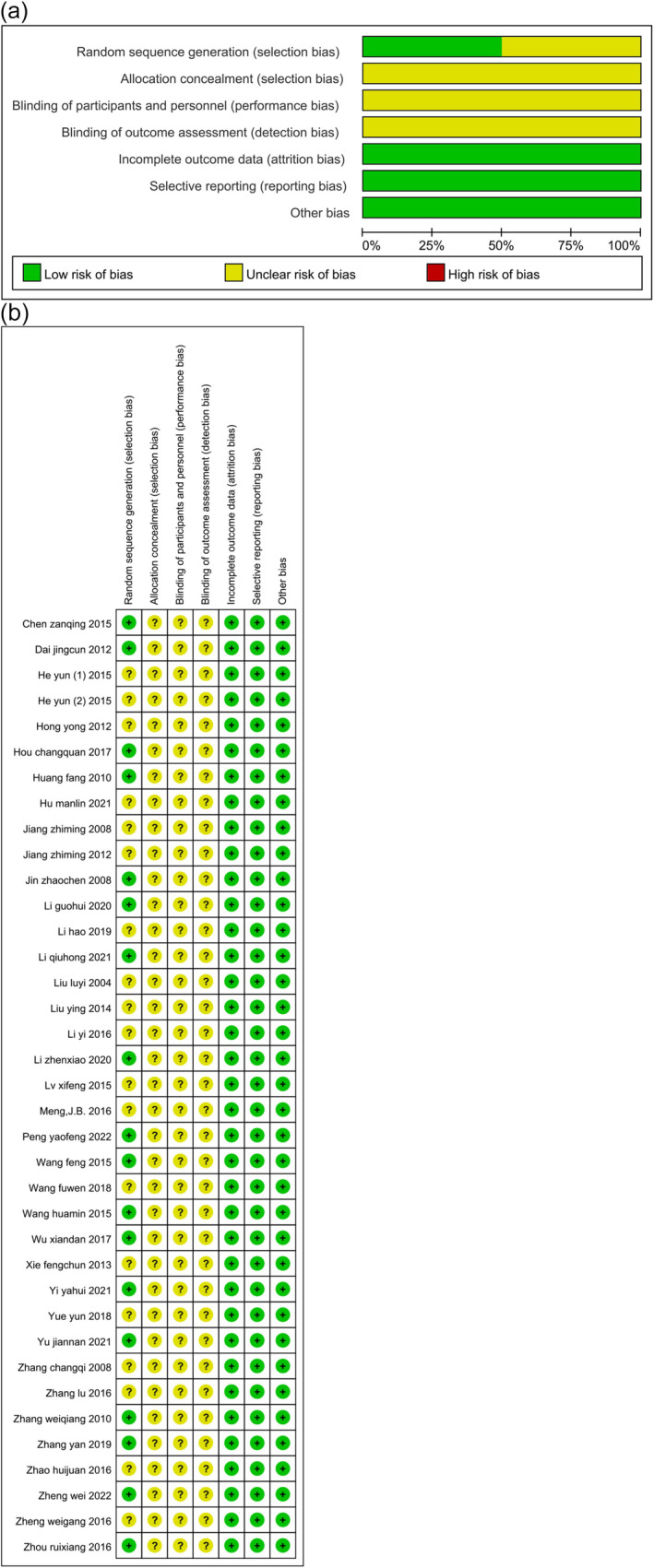
Quality assessment of included studies (a) Risk of bias graph. (b) Risk of bias summary.

The two evaluators reached highly consistent findings upon reviewing the studies. First, half of the studies (*n* = 18) reported the utilization of random sequences, yet none explicitly specified the application of allocation concealment. Second, none of the studies indicated the implementation of blind methods. Lastly, all studies presented comprehensive data, with no instances of selective reporting or other potential biases. Figure 2 visually represents the risk of bias.

## 4 Outcome Indicators

### 4.1 Main Outcome indicator

#### 4.1.1 Mortality Rate

**FIGURE 3:**
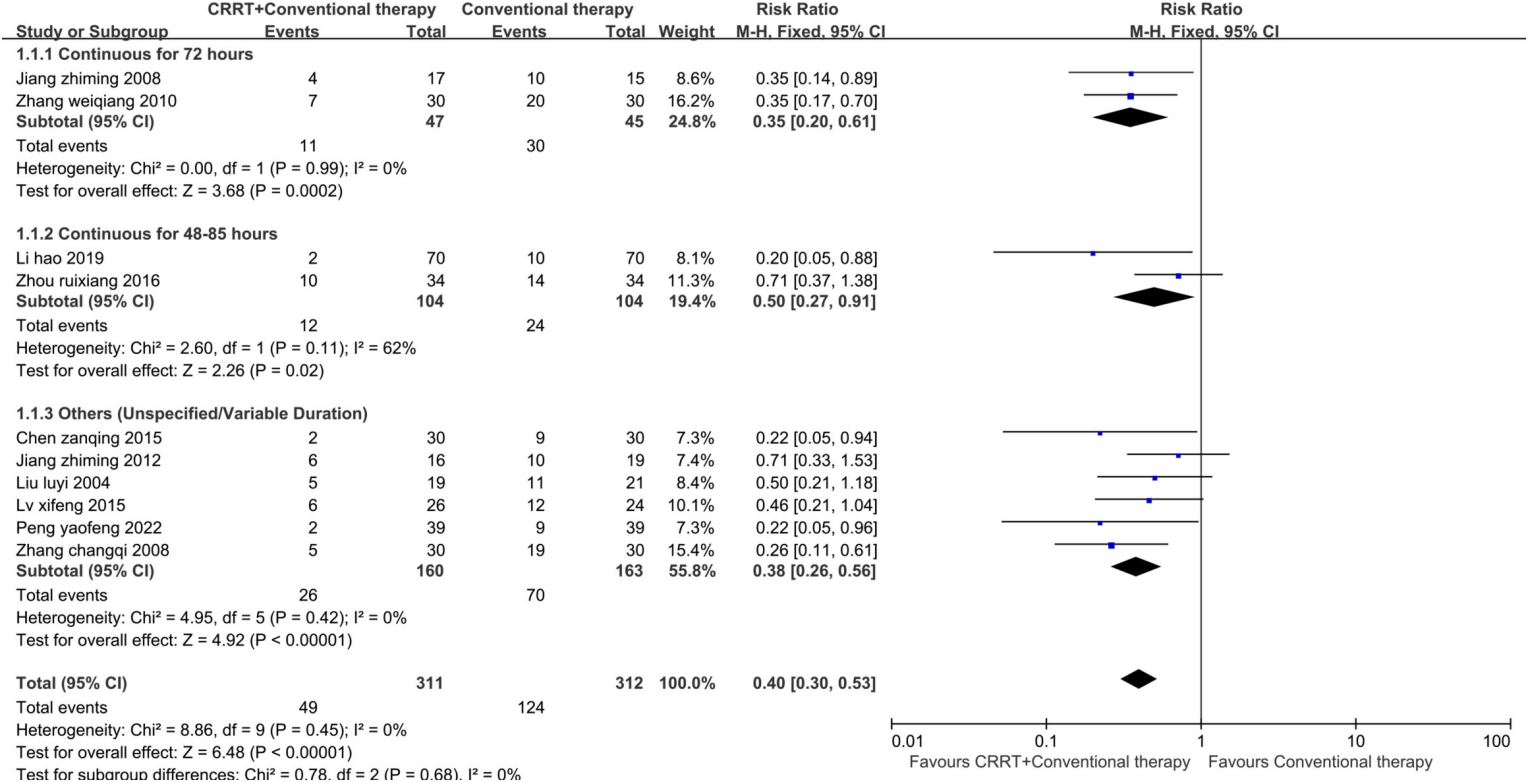
Forest plot of mortality rate.

Ten studies reported the mortality rate. Among these studies, one attributed the causes entirely to pancreatitis, another study solely to sepsis, while the remaining eight studies reported various critical conditions as causes. None of the 10 studies reported whether AKI was present. The results: *I* ^2^ = 0%; RR: 0.40; 95% CI: 0.30–0.53; *p* < 0.01 (fixed‐effects model) (Figure 3), which suggested that for ARDS, using CRRT in addition to conventional therapy can reduce the mortality rate. Two studies had a CRRT duration of 72 h, while another two studies had a CRRT duration ranging from 48 to 85 h. The remaining studies were categorized under unspecified/variable duration due to unreported or inconsistent CRRT durations. Subgroup analysis showed that CRRT reduced mortality in all three subgroups. The *p* value of Egger's test was 0.027 < 0.05, demonstrating publication bias. The final correction value was RR = 0.43, 95% CI: 0.33 to 0.57, *p* < 0.01, when using the trim and fill method, consistent with the previous results, showing that the finding was robust.

### 4.2 Secondary Outcome Indicators

**FIGURE 4:**
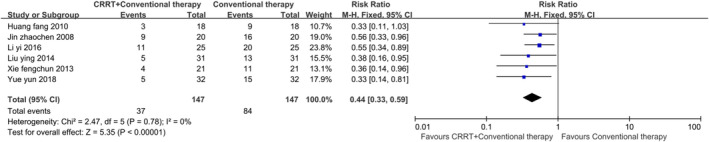
Forest plot of incidence of VAP.

**FIGURE 5:**
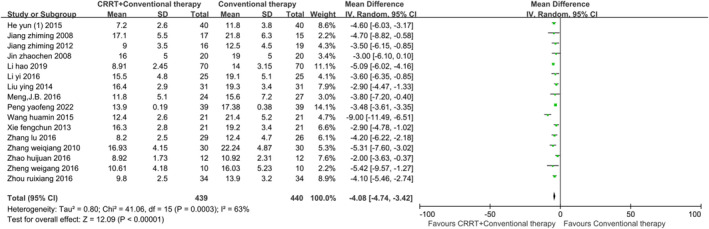
Forest plot of ICU length of stay.

**FIGURE 6:**
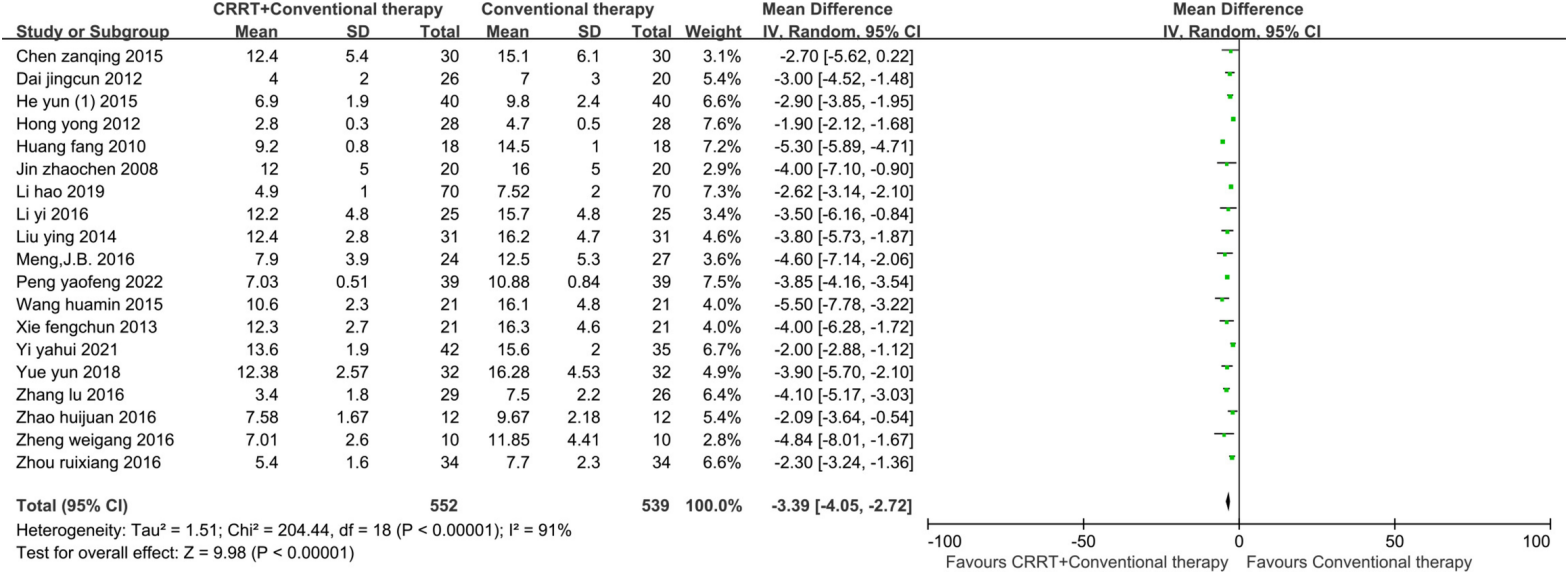
Forest plot of mechanical ventilation time.

The secondary outcome indicators comprised 19 items, including the incidence of VAP, ICU length of stay, mechanical ventilation time, EVLWI at 72 h, PaO_2_ at 72 h and measurements at different time intervals: OI, APACHE II score, TNF‐α, and IL‐6. Statistical analysis indicated that using CRRT in addition to conventional therapy can improve the above indicators in ARDS patients (*p* < 0.01). Egger's test revealed publication bias in two indicators. After applying the trim‐and‐fill method, the corrected data were consistent with or showed minimal differences from the original data, demonstrating the robustness of the meta‐analysis. Figures 4, 5, 6 depict the forest plots for the incidence of VAP, ICU length of stay, and mechanical ventilation time. Forest plots for the remaining secondary outcome indicators can be found in Data S4–S19.

### 4.3 Sensitivity Analysis

After systematically excluding each study individually for each outcome indicator, there were no significant alterations in the results. The statistical significance of the findings remained, underscoring the persistent stability and robustness of the results in this meta‐analysis.

All outcome indicators and their statistical analysis data can be found in Table 3.

**TABLE 3:** The summary of outcome indicators.

Sequence number	Outcome indicators	Initial data analysis								The *p* of Egger's test	Publication bias	After the correction of trim‐and‐fill method						Sensitivity analysis
		Number of studies	RR	WMD	95% CI	*I* ^2^	Effect model	*p*	Statistically significant			Number of studies	RR	WMD	95% CI	*p*	Robust	
a	Mortality rate	10	0.40	NA	0.30, 0.53	0%	Fixed	< 0.01	√	0.027	√	10	0.43	NA	0.33, 0.57	< 0.01	√	√
b	Incidence of VAP	6	0.44	NA	0.33, 0.59	0%	Fixed	< 0.01	√	0.002	√	6	0.47	NA	0.35, 0.63	< 0.01	√	√
c	ICU length of stay (days)	16	NA	−4.08	−4.74, −3.42	63%	Random	< 0.01	√	0.117	×	NA	NA	NA	NA	NA	NA	√
d	Mechanical ventilation time (days)	19	NA	−3.39	−4.05, −2.72	91%	Random	< 0.01	√	0.158	×	NA	NA	NA	NA	NA	NA	√
e1	OI at 24 h	8	NA	26.71	18.44, 34.97	68%	Random	< 0.01	√	0.566	×	NA	NA	NA	NA	NA	NA	√
e2	OI at 48 h	6	NA	35.80	25.79, 45.80	56%	Random	< 0.01	√	0.283	×	NA	NA	NA	NA	NA	NA	√
e3	OI at 72 h	14	NA	48.77	29.91, 67.63	98%	Random	< 0.01	√	0.104	×	NA	NA	NA	NA	NA	NA	√
e4	OI at 7 d	3	NA	31.76	16.73, 46.78	75%	Random	< 0.01	√	0.669	×	NA	NA	NA	NA	NA	NA	√
f	PaO_2_ at 72 h	3	NA	7.42	4.50, 10.33	79%	Random	< 0.01	√	0.264	×	NA	NA	NA	NA	NA	NA	√
g1	APACHE II score at 24 h	8	NA	−3.07	−3.45, −2.69	40%	Fixed	< 0.01	√	0.269	×	NA	NA	NA	NA	NA	NA	√
g2	APACHE II score at 48 h	5	NA	−3.48	−5.25, −1.70	71%	Random	< 0.01	√	0.251	×	NA	NA	NA	NA	NA	NA	√
g3	APACHE II score at 72 h	11	NA	−3.77	−5.04, −2.50	81%	Random	< 0.01	√	0.429	×	NA	NA	NA	NA	NA	NA	√
g4	APACHE II score at 7 d	4	NA	−2.23	−2.80, −1.67	0%	Fixed	< 0.01	√	0.299	×	NA	NA	NA	NA	NA	NA	√
h	EVLWI at 72 h (ml/kg)	3	NA	−2.90	−5.54, −0.26	95%	Random	< 0.01	√	0.563	×	NA	NA	NA	NA	NA	NA	√
i1	TNF‐α at 24 h (ng/L)	4	NA	−40.76	−58.08, −23.44	95%	Random	< 0.01	√	0.625	×	NA	NA	NA	NA	NA	NA	√
i2	TNF‐α at 7 d (ng/L)	4	NA	−11.09	−16.14, −6.04	83%	Random	< 0.01	√	0.753	×	NA	NA	NA	NA	NA	NA	√
j1	IL‐6 at 24 h (ng/L)	6	NA	−50.96	−67.80, −34.12	87%	Random	< 0.01	√	0.296	×	NA	NA	NA	NA	NA	NA	√
j2	IL‐6 at 48 h (ng/L)	4	NA	−46.80	−72.39, −21.20	92%	Random	< 0.01	√	0.531	×	NA	NA	NA	NA	NA	NA	√
j3	IL‐6 at 72 h (ng/L)	5	NA	−98.46	−137.70, −59.22	97%	Random	< 0.01	√	0.361	×	NA	NA	NA	NA	NA	NA	√
j4	IL‐6 at 7 d (ng/L)	4	NA	−24.60	−35.73, −13.47	88%	Random	< 0.01	√	0.016	√	4	−24.60	NA	−35.73, −13.47	< 0.01	√	√

*Note:* √ means yes.

Abbreviations: APACHE II, Acute Physiology and Chronic Health Evaluation II; EVLWI, extravascular lung water indexes; ICU, intensive care unit; IL‐6, interleukin‐6; NA, not applicable; OI, oxygenation index; PaO_2_, partial pressure of oxygen; TNF‐α, tumor necrosis factor‐α; VAP, ventilator‐associated pneumonia.

## 5 Discussion

First, this meta‐analysis suggests that adding CRRT to conventional therapy for ARDS may lower the mortality rate, incidence of VAP, ICU length of stay, and mechanical ventilation time. Second, it can improve OI during different time intervals and PaO_2_ at 72 h. Finally, it can also decrease EVLWI at 72 h and APACHE II score, TNF‐α, and IL‐6 at various time points.

The etiology of ARDS, such as sepsis and severe acute pancreatitis, involves triggering inflammatory responses that activate effector cells, leading to the release of a large number of cytokines and inflammatory mediators. This cascade of events results in a “cytokine storm,” initiating a series of chain reactions that ultimately lead to ARDS [8, 15]. Therefore, timely removal of circulating inflammatory mediators and blocking the progression of inflammatory responses are key aspects of treating ARDS [15]. Currently, the majority of cytokines or inflammatory mediators have been identified as the primary pathogenic factors of ARDS. Most of them are water‐soluble and belong to medium‐molecular‐weight peptides or proteins. They can be effectively removed through convection or filtration adsorption methods [8, 15]. CRRT is an effective method for removing these excessive cytokines. It can terminate the cascading inflammatory response by reducing the peak concentration of cytokines, thereby delaying or preventing the occurrence of ARDS and potential subsequent MODS. CRRT decreases the peak concentrations of pro‐inflammatory cytokines, establishing a dynamic equilibrium between pro‐inflammatory and anti‐inflammatory cytokines, thus mitigating widespread inflammatory responses in the lungs [15]. In this meta‐analysis, applying CRRT to ARDS patients led to a notable reduction in serum cytokines, such as TNF‐α and IL‐6, compared with those in the control group, aligning with theoretical predictions.

The Fluid and Catheter Treatment Trial (FACTT) indicates that a conservative fluid management strategy for patients with ARDS can reduce mechanical ventilation time and ICU length of stay and improve lung function. Still, it does not impact the mortality rate [59]. A recent study performed a subgroup analysis of patients from FACTT, identifying two unique ARDS phenotypes with differing reactions to fluid management approaches. The high‐inflammatory phenotype, marked by increased inflammatory cytokines, showed a greater mortality rate when assigned to a conservative fluid strategy. In contrast, the low‐inflammatory phenotype demonstrated a reduced mortality rate [60]. Consequently, different fluid management approaches may be required for distinct ARDS subtypes. ARDS patients may require adopting a conservative fluid strategy to reduce mortality rate. The study suggests that, compared with the high‐inflammatory subtype (23%), most ARDS patients are classified as the low‐inflammatory subtype (73%). Although the 2023 guidelines from the European Society of Intensive Care Medicine (ESICM) for fluid management in ARDS do not offer specific recommendations, the 2021 guidelines by the Japanese Respiratory Society (JRS) and the 2019 guidelines from the Intensive Care Society (ICS) in the United Kingdom both advocate for adopting a conservative fluid management approach for ARDS [61, 62, 63]. Meanwhile, the guidelines for the diagnosis of adult acute respiratory distress syndrome (ARDS) in China and nonmechanical ventilation treatment (2023) summarized 24 Chinese RCTs and 3 English RCTs and found that conservative fluid strategy compared with liberal fluid strategy can reduce mortality in ARDS patients. Therefore, it is recommended to use a conservative fluid strategy for ARDS patients [64]. At present, numerous clinicians target a negative fluid balance in managing fluid for ARDS patients, a strategy that seems advantageous for most individuals with ARDS [65].

Clinical evidence indicates that despite vigilant monitoring, most patients still undergo a positive fluid balance during the early stages of ARDS. This trend predicts longer mechanical ventilation durations, extended ICU and hospital stays, and a heightened mortality rate [66, 67]. Furthermore, increased static fluid pressure is frequently observed in patients with ARDS and is linked to a greater risk of mortality [67]. The fluid balance of ARDS patients is influenced by various factors, especially when they require fluid resuscitation in a state of shock. The fundamental treatment for ARDS patients is mechanical ventilation, and the positive pressure in the airways significantly contributes to a fluid‐positive balance. Fluid retention is an unavoidable consequence of employing positive‐pressure mechanical ventilation [67]. The mechanisms behind the negative impact of fluid overload on the prognosis of ARDS patients are not fully elucidated. In ARDS patients, the compromised function of the alveolar‐capillary barrier leads to a defective fluid clearance process [13]. Excessive fluid administration may result in increased pulmonary edema due to this impaired fluid clearance. The augmentation of pulmonary edema leads to reduced lung compliance and increased respiratory work, causing hypoxemia. Pulmonary edema also induces intrapulmonary shunting, leading to compression of pulmonary vessels and resulting in pulmonary hypertension [66]. Increasing evidence indicates that conservative fluid strategies may be associated with better outcomes in ARDS [68]. Furthermore, a study suggests that ARDS patients must clear alveolar edema to survive [69]. Early application of CRRT can play a crucial role in addressing dehydration, aiding in alleviating pulmonary edema and improving lung function [70]. EVLWI, associated with pulmonary edema, is also an independent predictor of ARDS mortality [71]. CRRT can reduce static hydrostatic pressure within the pulmonary circulation, decreasing EVLWI [72]. Based on the principles mentioned above, employing CRRT for managing ARDS patients may lead to better outcomes by achieving a negative fluid balance, which could be advantageous for most of them.

CRRT can rapidly eliminate excess heat from the bodies of ARDS patients, leading to a reduction in basal metabolic rate. This can lower oxygen consumption and thereby improve the OI [70, 73]. In addition, CRRT can enhance the antigen‐presenting and phagocytic capabilities of monocytes, neutrophils, and lymphocytes, thereby improving the body's immune function [65, 70, 74]. Our meta‐analysis identified improvements in various indicators for ARDS patients with CRRT, consistent with the abovementioned theories.

Our meta‐analysis searched multiple databases, providing the first comprehensive analysis of the effects of CRRT on ARDS patients with various outcome measures. We included grey literature, incorporating conference abstracts and master's thesis, to minimize publication bias. Employing the trim‐and‐fill method and sensitivity analysis, we enhanced the robustness of the meta‐analysis results.

The limitations of this meta‐analysis are as follows: (1) despite the inclusion of one English study, all the research was conducted in mainland China, and all were single‐center trials, leading to inevitable bias; (2) none of the included literature specified whether allocation concealment and blinding were employed, resulting in low‐quality literature; (3) CRRT is not a specific technique, and variations in filter type, mode, initiation time, blood flow rate, replacement fluid rate, ultrafiltration rate, treatment duration and frequency, anticoagulant type, and fluid balance may contribute to varying degrees of clinical heterogeneity. Similarly, differences in conventional therapies used in the control group (such as ventilation mode and positive end‐expiratory pressure) may exist, particularly in the context of different primary treatments for varying etiologies of ARDS. Additionally, almost all the included studies did not report the safety and side effects of CRRT, so this analysis could not be performed in our study; (4) due to insufficient information, subgroup analyses based on the presence of AKI in ARDS patients and the etiology of ARDS could not be conducted, and meta‐regression to analyze the sources of heterogeneity for outcomes with substantial heterogeneity was not possible. Using a random‐effects model for highly heterogeneous results may reduce the efficiency of the meta‐analysis; (5) if ARDS patients experience concomitant AKI, the mortality rate significantly increases. In a trial for ARDS, the mortality rate for patients with both AKI and ARDS was nearly twice that of patients with ARDS alone [75]. Most studies in this meta‐analysis did not specify whether ARDS patients also had AKI, so while the meta‐analysis results suggest that CRRT can reduce the mortality rate, the extent of this reduction may be biased. Moreover, ARDS is a highly heterogeneous clinical syndrome, and variations in its severity, different underlying diseases, and the presence of high or low inflammatory phenotypes could all influence patients' ultimate outcomes, thus affecting the results of the meta‐analysis.

Given the above limitations, we are unable to determine the conditions for initiating CRRT to achieve the best therapeutic response, such as reducing mortality and improving oxygenation index. This meta‐analysis, however, highlights the potential of CRRT as an adjunctive therapy in ARDS, showing promising reductions in mortality, improved oxygenation, and shorter ICU stays. Despite these findings, the study underscores significant gaps in current research, particularly regarding the optimal initiation timing and duration of CRRT needed to maximize benefits and minimize side effects. These limitations underscore the urgent need for well‐designed, multicenter trials to establish precise initiation conditions and duration thresholds for CRRT in ARDS patients. As intensive care specialists continue to manage this complex condition, this study calls for a careful balance of therapeutic interventions and advocates further research to refine CRRT protocols for improved patient outcomes.

## 6 Conclusion

Low‐quality evidence suggests that compared with conventional therapy alone, the use of CRRT may be associated with a lower mortality rate, the incidence of VAP, ICU length of stay, mechanical ventilation time, EVLWI, APACHE II score, TNF‐α, and IL‐6 and may be related to better respiratory function. CRRT may be beneficial for ARDS patients. Future multicenter, well‐designed, high‐quality RCTs are needed to substantiate these findings.

## Author Contributions

Siyao Zeng and Hongliang Wang were involved in formulating the study concept. Siyao Zeng, Shanpeng Cui, Yue Li, and Zhipeng Yao carried out the review of literature and extraction of data. Yunlong Li, Junbo Zheng and Yang Cao performed the quality assessment. Siyao Zeng, Lianghe Wen, and Ming Li conducted the statistical analysis. All authors participated in the writing of the manuscript and approved the final manuscript for publication.

## Ethics Statement

An ethics statement is not applicable because this study is based exclusively on published literature.

## Conflicts of Interest

The authors declare no conflicts of interest.

## Supporting information


**Data S1** Search strategy.

**Data S2** Forest‐plot of OI at 24 h.

**Data S3** Forest‐plot of OI at 48 h.

**Data S4** Forest‐plot of OI at 72 h.

**Data S5** Forest‐plot of OI at 7 d.

**Data S6** Forest‐plot of PaO_2_ at 72 h.

**Data S7** Forest‐plot of APACHE II score at 24 h.

**Data S8** Forest‐plot of APACHE II score at 48 h.

**Data S9** Forest‐plot of APACHE II score at 72 h.

**Data S10** Forest‐plot of APACHE II score at 7 d.

**Data S11** Forest‐plot of EVLWI at 72 h.

**Data S12** Forest‐plot of TNF‐α at 24 h.

**Data S13** Forest‐plot of TNF‐α at 7 d.

**Data S14** Forest‐plot of IL‐6 at 24 h.

**Data S15** Forest‐plot of IL‐6 at 48 h.

**Data S16** Forest‐plot of IL‐6 at 72 h.

**Data S17** Forest‐plot of IL‐6 at 7 d.

**Data S18** PRISMA_2020_checklist.

## Data Availability

All data generated or analyzed during this study are included in this article and its supporting information. Further inquiries can be directed to the corresponding author.
