# Supplementary material for: New Insights on Continuous Renal Replacement Therapy for Acute Respiratory Distress Syndrome: A Systematic Review and Meta‐Analysis
Source: Clin Respir J. 2025 Jan 2;19(1):e70045. doi: 10.1111/crj.70045 (PMC11695202; doi:10.1111/crj.70045)
Supplement: Supplementary file 1 — Data S1 Search strategy. [file CRJ-19-e70045-s005.doc]

literature search

1、Chinese National Knowledge Infrastructure (CNKI)

疾病类型：

主题词：急性呼吸窘迫综合征

自由词：成人呼吸窘迫综合征、ARDS、急性肺损伤、休克肺

干预措施：

主题词：连续性肾脏替代治疗

自由词：连续性肾脏替代疗法、持续性肾脏替代治疗、持续性肾脏替代疗法、连续性血液净化、持续性血液净化、连续性静脉-静脉血液滤过、连续性静脉-静脉血液透析、连续性静脉-静脉血液透析滤过、CRRT、CVVH、CVVHD、CVVHDF

研究方法：

主题词：随机对照 自由词：随机分配、随机、抽签法、随机数字表

SU%=急性呼吸窘迫综合征 OR SU%=成人呼吸窘迫综合征 OR SU%=ARDS OR SU%=急性肺损伤 OR SU%=休克肺

SU%=连续性肾脏替代治疗 OR SU%=连续性肾脏替代疗法 OR SU%=持续性肾脏替代治疗 OR SU%=持续性肾脏替代疗法 OR SU%=连续性血液净化 OR SU%=持续性血液净化 OR SU%=连续性静脉-静脉血液滤过 OR SU%=连续性静脉-静脉血液透析 OR SU%=连续性静脉-静脉血液透析滤过 OR SU%=CRRT OR SU%=CVVH OR SU%=CVVHD OR SU%=CVVHDF

AB%=随机对照 OR AB%=随机分配 OR AB%=随机 OR AB%=抽签法 OR AB%=随机数字表

检索式：(SU%=急性呼吸窘迫综合征 OR SU%=成人呼吸窘迫综合征 OR SU%=ARDS OR SU%=急性肺损伤 OR SU%=休克肺) AND (SU%=连续性肾脏替代治疗 OR SU%=连续性肾脏替代疗法 OR SU%=持续性肾脏替代治疗 OR SU%=持续性肾脏替代疗法 OR SU%=连续性血液净化 OR SU%=持续性血液净化 OR SU%=连续性静脉-静脉血液滤过 OR SU%=连续性静脉-静脉血液透析 OR SU%=连续性静脉-静脉血液透析滤过 OR SU%=CRRT OR SU%=CVVH OR SU%=CVVHD OR SU%=CVVHDF ) AND (AB%=随机对照 OR AB%=随机分配 OR AB%=随机 OR AB%=抽签法 OR AB%=随机数字表)

1. WanFang

检索策略(检索式)：主题:(急性呼吸窘迫综合征 or 成人呼吸窘迫综合征 or ARDS or 急性肺损伤 or 休克肺) and 主题:(连续性肾脏替代治疗 or 连续性肾脏替代疗法 or 持续性肾脏替代治疗 or 持续性肾脏替代疗法 or 连续性血液净化 or 持续性血液净化 or连续性静脉-静脉血液滤过 or 连续性静脉-静脉血液透析 or 连续性静脉-静脉血液透析滤过 or CRRT or CVVH or CVVHD or CVVHDF) and 摘要:(随机对照 or 随机分配 or 随机 or 抽签法 or 随机数字表)

1. China Science and Technology Journal Database (VIP)

急性呼吸窘迫综合征+成人呼吸窘迫综合征+ ARDS+急性肺损伤+休克肺

连续性肾脏替代治疗+连续性肾脏替代疗法+持续性肾脏替代治疗+持续性肾脏替代疗法+连续性血液净化+持续性血液净化+连续性静脉-静脉血液滤过+连续性静脉-静脉血液透析+连续性静脉-静脉血液透析滤过+CRRT+CVVH+CVVHD+CVVHDF

随机对照+随机分配+随机+抽签法+随机数字表

(M=急性呼吸窘迫综合征 OR M=成人呼吸窘迫综合征 OR M=ARDS OR M=急性肺损伤 OR M=休克肺) AND (M=连续性肾脏替代治疗 OR M=连续性肾脏替代疗法 OR M=持续性肾脏替代治疗 OR M=持续性肾脏替代疗法 OR M=连续性血液净化 OR M=持续性血液净化 OR M=连续性静脉-静脉血液滤过 OR M=连续性静脉-静脉血液透析 OR M=连续性静脉-静脉血液透析滤过 OR M=CRRT OR M=CVVH OR M=CVVHD OR M=CVVHDF) AND (R=随机对照 OR R=随机分配 OR R=随机 OR R=抽签法 OR R=随机数字表)

1. Chinese Biomedical literature（CBM）

检索策略(检索式)：("随机数字表"[摘要] OR "抽签法"[摘要] OR "随机"[摘要] OR ("随机分配"[摘要] OR "随机化"[摘要]) OR "随机对照"[摘要]) AND ("急性呼吸窘迫综合征"[标题] OR "成人呼吸窘迫综合征"[标题] OR "ARDS"[标题] OR "急性肺损伤"[标题] OR "休克肺"[标题]) AND ("连续性肾脏替代治疗"[标题] OR "连续性肾脏替代疗法"[标题] OR "持续性肾脏替代治疗"[标题] OR "持续性肾脏替代疗法"[标题] OR "连续性血液净化"[标题] OR "持续性血液净化"[标题] OR "连续性静脉-静脉血液滤过"[标题] OR "连续性静脉-静脉血液透析"[标题] OR "连续性静脉-静脉血液透析滤过"[标题] OR "CRRT"[标题] OR "CVVH"[标题] OR "CVVHD"[标题] OR "CVVHDF"[标题])

1. Duxiu

(T=急性呼吸窘迫综合征|S=急性呼吸窘迫综合征|T=成人呼吸窘迫综合征|S=成人呼吸窘迫综合征|T=ARDS|S=ARDS|T=急性肺损伤|S=急性肺损伤|T=休克肺|S=休克肺)*(T=连续性肾脏替代治疗|S=连续性肾脏替代治疗|T=连续性肾脏替代疗法|S=连续性肾脏替代疗法|T=持续性肾脏替代治疗|S=持续性肾脏替代治疗|T=持续性肾脏替代疗法|S=持续性肾脏替代疗法|T=连续性血液净化|S=连续性血液净化|T=持续性血液净化|S=持续性血液净化|T=连续性静脉-静脉血液滤过|S=连续性静脉-静脉血液滤过|T=连续性静脉-静脉血液透析|S=连续性静脉-静脉血液透析|T=连续性静脉-静脉血液透析滤过|S=连续性静脉-静脉血液透析滤过|T=CRRT|S=CRRT|T=CVVH|S=CVVH|T=CVVHD|S=CVVHD|T=CVVHDF|S=CVVHDF)*(T=随机对照|S=随机对照|T=随机分配|S=随机分配|T=随机|S=随机|T=抽签法|S=抽签法|T=随机数字表|S=随机数字表)

6、Pubmed

Disease type

Subject Word：Respiratory Distress Syndrome

random word：

Respiratory Distress Syndromes

Shock Lung

Acute Respiratory Distress Syndrome

ARDS

Human ARDS

Adult Respiratory Distress Syndrom

Intervention measures:

Subject Word：Continuous renal replacement therapy

random word：

Continuous RRT

Continuous Renal Replacement Procedure

CRRT

Continuous Venovenous Hemofiltration

CVVH

Continuous Veno Venous Hemodialysis

CVVHD

Continuous Veno Venous Hemodiafiltration

CVVHDF

Continuous blood purification

CBP

Extracorporeal Blood Purification

research method：

randomized controlled trial[Publication Type] OR

randomized[Title/Abstract] OR

placebo[Title/Abstract]

Search: ****((((((((Respiratory Distress Syndrome[Title/Abstract]) OR (Respiratory Distress Syndromes[Title/Abstract])) OR (Shock Lung[Title/Abstract])) OR (Acute Respiratory Distress Syndrome[Title/Abstract])) OR (ARDS[Title/Abstract])) OR (Human ARDS[Title/Abstract])) OR (Adult Respiratory Distress Syndrom[Title/Abstract])) AND (((((((((((((Continuous renal replacement therapy[Title/Abstract]) OR (Continuous RRT[Title/Abstract])) OR (Continuous Renal Replacement Procedure[Title/Abstract])) OR (CRRT[Title/Abstract])) OR (Continuous Venovenous Hemofiltration[Title/Abstract])) OR (CVVH[Title/Abstract])) OR (Continuous Veno Venous Hemodialysis[Title/Abstract])) OR (CVVHD[Title/Abstract])) OR (Continuous Veno Venous Hemodiafiltration[Title/Abstract])) OR (CVVHDF[Title/Abstract])) OR (Continuous blood purification[Title/Abstract])) OR (CBP[Title/Abstract])) OR (Extracorporeal Blood Purification[Title/Abstract]))) AND (randomized controlled trial[Publication Type] OR randomized[Title/Abstract] OR placebo[Title/Abstract])****

7、Embase

'Respiratory Distress Syndrome':ab,ti OR 'Respiratory Distress Syndromes':ab,ti OR 'Shock Lung':ab,ti OR 'Acute Respiratory Distress Syndrome':ab,ti OR 'ARDS':ab,ti OR 'Human ARDS':ab,ti OR 'Adult Respiratory Distress Syndrom':ab,ti

'Continuous renal replacement therapy':ab,ti OR 'Continuous RRT':ab,ti OR 'Continuous Renal Replacement Procedure':ab,ti OR 'CRRT':ab,ti OR 'Continuous Venovenous Hemofiltration':ab,ti OR 'CVVH':ab,ti OR 'Continuous Veno Venous Hemodialysis':ab,ti OR 'CVVHD':ab,ti OR 'Continuous Veno Venous Hemodiafiltration':ab,ti OR 'CVVHDF':ab,ti OR 'Continuous blood purification':ab,ti OR 'CBP ':ab,ti OR 'Extracorporeal Blood Purification':ab,ti

'randomized controlled trial':ab,ti or 'randomized':ab,ti or 'placebo':ab,ti

Embase

Session Results

.......................................................

No. Query Results Results Date

#4. #1 AND #2 AND #3 15 28 Nov 2023

#3. 'randomized controlled trial':ab,ti OR 1,156,733 28 Nov 2023

'randomized':ab,ti OR 'placebo':ab,ti

#2. 'continuous renal replacement therapy':ab,ti OR 20,672 28 Nov 2023

'continuous rrt':ab,ti OR 'continuous renal

replacement procedure':ab,ti OR 'crrt':ab,ti OR

'continuous venovenous hemofiltration':ab,ti OR

'cvvh':ab,ti OR 'continuous veno venous

hemodialysis':ab,ti OR 'cvvhd':ab,ti OR

'continuous veno venous hemodiafiltration':ab,ti

OR 'cvvhdf':ab,ti OR 'continuous blood

purification':ab,ti OR 'cbp':ab,ti OR

'extracorporeal blood purification':ab,ti

#1. 'respiratory distress syndrome':ab,ti OR 60,077 28 Nov 2023

'respiratory distress syndromes':ab,ti OR 'shock

lung':ab,ti OR 'acute respiratory distress

syndrome':ab,ti OR 'ards':ab,ti OR 'human

ards':ab,ti OR 'adult respiratory distress

syndrom':ab,ti

.......................................................

8、Cochrane

(Respiratory Distress Syndrome):ti,ab,kw or (Respiratory Distress Syndromes):ti,ab,kw or (Shock Lung):ti,ab,kw or (Acute Respiratory Distress Syndrome):ti,ab,kw or (ARDS):ti,ab,kw or (Human ARDS):ti,ab,kw or (Adult Respiratory Distress Syndrom):ti,ab,kw

(Continuous renal replacement therapy):ti,ab,kw or (Continuous RRT):ti,ab,kw or (Continuous Renal Replacement Procedure):ti,ab,kw or (CRRT):ti,ab,kw or (Continuous Venovenous Hemofiltration):ti,ab,kw or (CVVH):ti,ab,kw or (Continuous Veno Venous Hemodialysis):ti,ab,kw or (CVVHD):ti,ab,kw or (Continuous Veno Venous Hemodiafiltration):ti,ab,kw or (CVVHDF):ti,ab,kw or (Continuous blood purification):ti,ab,kw or (CBP ):ti,ab,kw or (Extracorporeal Blood Purification):ti,ab,kw

(randomized controlled trial):ab or (randomized):ab or (placebo):ab

Search Name:

Date Run: 28/11/2023 10:56:56

Comment:

ID Search Hits

#1 (Respiratory Distress Syndrome):ti,ab,kw or (Respiratory Distress Syndromes):ti,ab,kw or (Shock Lung):ti,ab,kw or (Acute Respiratory Distress Syndrome):ti,ab,kw or (ARDS):ti,ab,kw or (Human ARDS):ti,ab,kw or (Adult Respiratory Distress Syndrom):ti,ab,kw 8280

#2 (Continuous renal replacement therapy):ti,ab,kw or (Continuous RRT):ti,ab,kw or (Continuous Renal Replacement Procedure):ti,ab,kw or (CRRT):ti,ab,kw or (Continuous Venovenous Hemofiltration):ti,ab,kw or (CVVH):ti,ab,kw or (Continuous Veno Venous Hemodialysis):ti,ab,kw or (CVVHD):ti,ab,kw or (Continuous Veno Venous Hemodiafiltration):ti,ab,kw or (CVVHDF):ti,ab,kw or (Continuous blood purification):ti,ab,kw or (CBP ):ti,ab,kw or (Extracorporeal Blood Purification):ti,ab,kw 1964

#3 (randomized controlled trial):ab or (randomized):ab or (placebo):ab 956378

#4 #1 and #2 and #3 48

9、OVID

(Respiratory Distress Syndrome or Respiratory Distress Syndromes or Shock Lung or Acute Respiratory Distress Syndrome or ARDS or Human ARDS or Adult Respiratory Distress Syndrom).ti,ab,kw

(continuous renal replacement therapy or Continuous RRT or Continuous Renal Replacement Procedure or CRRT or Continuous Venovenous Hemofiltration or CVVH or Continuous Veno Venous Hemodialysis or CVVHD or Continuous Veno Venous Hemodiafiltration or CVVHDF or Continuous blood purification or CBP or Extracorporeal Blood Purification).ti,ab,kw

(randomized controlled trial or randomized or placebo).ti,ab,kw

Ovid MEDLINE(R) ALL <1946 to November 27, 2023>

1 (Respiratory Distress Syndrome or Respiratory Distress Syndromes or Shock Lung or Acute Respiratory Distress Syndrome or ARDS or Human ARDS or Adult Respiratory Distress Syndrom).ti,ab,kw. 43104

2 (continuous renal replacement therapy or Continuous RRT or Continuous Renal Replacement Procedure or CRRT or Continuous Venovenous Hemofiltration or CVVH or Continuous Veno Venous Hemodialysis or CVVHD or Continuous Veno Venous Hemodiafiltration or CVVHDF or Continuous blood purification or CBP or Extracorporeal Blood Purification).ti,ab,kw. 13238

3 (randomized controlled trial or randomized or placebo).ti,ab,kw. 809996

4 1 and 2 and 3 10

10、Web of Sciense

Respiratory Distress Syndrome or Respiratory Distress Syndromes or Shock Lung or Acute Respiratory Distress Syndrome or ARDS or Human ARDS or Adult Respiratory Distress Syndrom

Continuous renal replacement therapy or Continuous RRT or Continuous Renal Replacement Procedure or CRRT or Continuous Venovenous Hemofiltration or CVVH or Continuous Veno Venous Hemodialysis or CVVHD or Continuous Veno Venous Hemodiafiltration or CVVHDF or Continuous blood purification or CBP or Extracorporeal Blood Purification

randomized controlled trial or randomized or placebo

# Web of Science Search Strategy (v0.1)

# Database: Web of Science Core Collection

# Entitlements:

- WOS.IC: 1993 to 2023

- WOS.CCR: 1985 to 2023

- WOS.SCI: 1975 to 2023

- WOS.AHCI: 1975 to 2023

- WOS.BHCI: 2005 to 2023

- WOS.BSCI: 2005 to 2023

- WOS.ESCI: 2018 to 2023

- WOS.ISTP: 1990 to 2023

- WOS.SSCI: 1965 to 2023

- WOS.ISSHP: 1990 to 2023

# Searches:

1: Respiratory Distress Syndrome or Respiratory Distress Syndromes or Shock Lung or Acute Respiratory Distress Syndrome or ARDS or Human ARDS or Adult Respiratory Distress Syndrom (Topic) AND Continuous renal replacement therapy or Continuous RRT or Continuous Renal Replacement Procedure or CRRT or Continuous Venovenous Hemofiltration or CVVH or Continuous Veno Venous Hemodialysis or CVVHD or Continuous Veno Venous Hemodiafiltration or CVVHDF or Continuous blood purification or CBP or Extracorporeal Blood Purification (Topic) AND randomized controlled trial or randomized or placebo (Abstract) Date Run: Tue Nov 28 2023 19:10:22 GMT+0800 (GMT+08:00) Results: 31

11、Scopus

"Respiratory Distress Syndrome" OR "Respiratory Distress Syndromes" OR "Shock Lung" OR "Acute Respiratory Distress Syndrome" OR "ARDS" OR "Human ARDS" OR "Adult Respiratory Distress Syndrom"

"continuous renal replacement therapy" OR "Continuous RRT" OR "Continuous Renal Replacement Procedure" OR "CRRT" OR "Continuous Venovenous Hemofiltration" OR "CVVH" OR "Continuous Veno Venous Hemodialysis" OR "CVVHD" OR "Continuous Veno Venous Hemodiafiltration" OR "CVVHDF" OR "Continuous blood purification" OR "CBP " OR "Extracorporeal Blood Purification"

"randomized controlled trial" OR "randomized" OR "placebo"

( TITLE-ABS-KEY ( "Respiratory Distress Syndrome" OR "Respiratory Distress Syndromes" OR "Shock Lung" OR "Acute Respiratory Distress Syndrome" OR "ARDS" OR "Human ARDS" OR "Adult Respiratory Distress Syndrom" ) AND TITLE-ABS-KEY ( "continuous renal replacement therapy" OR "Continuous RRT" OR "Continuous Renal Replacement Procedure" OR "CRRT" OR "Continuous Venovenous Hemofiltration" OR "CVVH" OR "Continuous Veno Venous Hemodialysis" OR "CVVHD" OR "Continuous Veno Venous Hemodiafiltration" OR "CVVHDF" OR "Continuous blood purification" OR "CBP " OR "Extracorporeal Blood Purification" ) AND ABS ( "randomized controlled trial" OR "randomized" OR "placebo" ) )

12、ProQuest

AB,TI(''Respiratory Distress Syndrome'' OR ''Respiratory Distress Syndromes'' OR ''Shock Lung'' OR ''Acute Respiratory Distress Syndrome'' OR ''ARDS'' OR ''Human ARDS'' OR ''Adult Respiratory Distress Syndrom'')

AB,TI(''Continuous renal replacement therapy'' OR ''Continuous RRT'' OR ''Continuous Renal Replacement Procedure'' OR ''CRRT'' OR ''Continuous Venovenous Hemofiltration'' OR ''CVVH'' OR ''Continuous Veno Venous Hemodialysis'' OR ''CVVHD'' OR ''Continuous Veno Venous Hemodiafiltration'' OR ''CVVHDF'' OR ''Continuous blood purification'' OR ''CBP'' OR ''Extracorporeal Blood Purification'')

AB,TI(''randomized controlled trial'' OR ''randomized'' OR ''placebo'')

检索策略

Set#: S1

Searched for: AB,TI(''Respiratory Distress Syndrome'' OR ''Respiratory Distress Syndromes'' OR ''Shock Lung'' OR ''Acute Respiratory Distress Syndrome'' OR ''ARDS'' OR ''Human ARDS'' OR ''Adult Respiratory Distress Syndrom'')

Databases: Acta Sanctorum, Acta Sanctorum, African American Poetry, African Writers Series, African Writers Series, Alt-PressWatch, American Periodicals, American Poetry, Annual Bibliography of English Language and Literature (ABELL), Archive Finder, Art & Architecture Archive, Arts Premium Collection, Bertolt Brechts Werke, Bibliografía de la Literatura Española, Black Abolitionist Papers, Black Abolitionist Papers, Black Studies Center, British Periodicals, C19: The Nineteenth Century Index, Canadian Poetry, Children's Magazine Archive, 1866-2020, Children's Magazine Archive, 1866-2020, Colonial Legacies: Empire & Commonwealth Periodicals, Colonial Legacies: Empire & Commonwealth Periodicals, Colonial State Papers, Colonial State Papers, Coronavirus Research Database, Country Life Archive, Country Life Archive, Die Deutsche Lyrik in Reclams Universal-Bibliothek, Digital National Security Archive, Documents on British Policy Overseas, Documents on British Policy Overseas, Early American Fiction 1789-1875, Early English Prose Fiction, Early Modern Books, Ebook Central, Economist Intelligence Unit Country Reports Archive, Economist Intelligence Unit Country Reports Archive, Education Magazine Archive, Education Magazine Archive, Eighteenth-Century Fiction, eLibrary, English Drama, English Poetry, Second Edition, Entertainment Industry Magazine Archive, Ethnic NewsWatch, GenderWatch, GeoRef, Gerritsen Women's History Collection of Aletta H. Jacobs, Gerritsen Women's History Collection of Aletta H. Jacobs, Goethes Werke, Health & Fitness Magazine Archive, Health & Fitness Magazine Archive, Health Research Premium Collection, Historic Literary Criticism, History Study Center, History Study Center, Humanities Index, John Johnson Collection: An Archive of Printed Ephemera, John Johnson Collection: An Archive of Printed Ephemera, Kafkas Werke, Latinx Thought and Culture: The NPR Archive, 1979-1990, Latinx Thought and Culture: The NPR Archive, 1979-1990, LGBT Magazine Archive, LGBT Magazine Archive Collection 1, Literature Online, Men’s Magazine Archive, Men’s Magazine Archive, News, Policy & Politics Magazine Archive (feat. Newsweek), News, Policy & Politics Magazine Archive (feat. Newsweek), Nineteenth-Century Fiction, Patrologia Latina, Patrologia Latina, Periodicals Archive Online, Periodicals Index Online, Philosopher's Index, Policy File Index, PRISMA Database, PRISMA Database, ProQuest Central, ProQuest Civil War Era, ProQuest Civil War Era, ProQuest Dissertations & Theses Global, ProQuest Historical Annual Reports, ProQuest Historical Newspapers: Atlanta Daily World, ProQuest Historical Newspapers: Atlanta Daily World, ProQuest Historical Newspapers: Calgary Herald, ProQuest Historical Newspapers: Calgary Herald, ProQuest Historical Newspapers: Chicago Defender, ProQuest Historical Newspapers: Chicago Defender, ProQuest Historical Newspapers: Chicago Defender, ProQuest Historical Newspapers: Chicago Defender, ProQuest Historical Newspapers: Chicago Tribune, ProQuest Historical Newspapers: Chicago Tribune, ProQuest Historical Newspapers: Chicago Tribune, ProQuest Historical Newspapers: Chinese Newspapers Collection, ProQuest Historical Newspapers: Chinese Newspapers Collection, ProQuest Historical Newspapers: Cleveland Call and Post, ProQuest Historical Newspapers: Cleveland Call and Post, ProQuest Historical Newspapers: Communist Historical Newspaper Collection, ProQuest Historical Newspapers: Communist Historical Newspaper Collection, ProQuest Historical Newspapers: Dayton Daily News, ProQuest Historical Newspapers: Dayton Daily News, ProQuest Historical Newspapers: Detroit Free Press, ProQuest Historical Newspapers: Detroit Free Press (1831-1922), ProQuest Historical Newspapers: Edmonton Journal, ProQuest Historical Newspapers: Edmonton Journal, ProQuest Historical Newspapers: Hartford Courant, ProQuest Historical Newspapers: Hartford Courant, ProQuest Historical Newspapers: Indianapolis Star, ProQuest Historical Newspapers: Indianapolis Star (1903-1922), ProQuest Historical Newspapers: Leader-Post, ProQuest Historical Newspapers: Leader-Post, ProQuest Historical Newspapers: Leftist Newspapers and Periodicals, ProQuest Historical Newspapers: Leftist Newspapers and Periodicals, ProQuest Historical Newspapers: Los Angeles Sentinel, ProQuest Historical Newspapers: Los Angeles Sentinel, ProQuest Historical Newspapers: Los Angeles Times, ProQuest Historical Newspapers: Los Angeles Times, ProQuest Historical Newspapers: Los Angeles Times, ProQuest Historical Newspapers: Louisville Courier Journal, ProQuest Historical Newspapers: Michigan Chronicle, ProQuest Historical Newspapers: Michigan Chronicle, ProQuest Historical Newspapers: Minneapolis Star Tribune, ProQuest Historical Newspapers: Minneapolis Star Tribune, ProQuest Historical Newspapers: Montreal Gazette, ProQuest Historical Newspapers: Montreal Gazette, ProQuest Historical Newspapers: Newsday, ProQuest Historical Newspapers: Newsday, ProQuest Historical Newspapers: New York Amsterdam News, ProQuest Historical Newspapers: New York Tribune, ProQuest Historical Newspapers: Norfolk Journal and Guide, ProQuest Historical Newspapers: Norfolk Journal and Guide, ProQuest Historical Newspapers: Ottawa Citizen, ProQuest Historical Newspapers: Ottawa Citizen, ProQuest Historical Newspapers: Philadelphia Tribune, ProQuest Historical Newspapers: Philadelphia Tribune, ProQuest Historical Newspapers: Pittsburgh Courier, ProQuest Historical Newspapers: Pittsburgh Courier, ProQuest Historical Newspapers: Pittsburgh Post-Gazette, ProQuest Historical Newspapers: Pittsburgh Post-Gazette, ProQuest Historical Newspapers: San Francisco Chronicle, ProQuest Historical Newspapers: San Francisco Chronicle, ProQuest Historical Newspapers: Saskatoon Star-Phoenix, ProQuest Historical Newspapers: Saskatoon Star-Phoenix, ProQuest Historical Newspapers: South China Morning Post, ProQuest Historical Newspapers: South China Morning Post, ProQuest Historical Newspapers: St. Louis Post Dispatch, ProQuest Historical Newspapers: St. Louis Post-Dispatch (1874-1922), ProQuest Historical Newspapers: St. Petersburg Times / Tampa Bay Times, ProQuest Historical Newspapers: St. Petersburg Times / Tampa Bay Times, ProQuest Historical Newspapers: The American Hebrew & Jewish Messenger, ProQuest Historical Newspapers: The American Hebrew & Jewish Messenger, ProQuest Historical Newspapers: The American Israelite, ProQuest Historical Newspapers: The American Israelite, ProQuest Historical Newspapers: The Arizona Republican, ProQuest Historical Newspapers: The Arizona Republican (1890-1922), ProQuest Historical Newspapers: The Atlanta Constitution, ProQuest Historical Newspapers: The Atlanta Constitution, ProQuest Historical Newspapers: The Atlanta Constitution, ProQuest Historical Newspapers: The Baltimore Afro-American, ProQuest Historical Newspapers: The Baltimore Afro-American, ProQuest Historical Newspapers: The Baltimore Sun, ProQuest Historical Newspapers: The Baltimore Sun, ProQuest Historical Newspapers: The Boston Globe, ProQuest Historical Newspapers: The Boston Globe, ProQuest Historical Newspapers: The Christian Science Monitor, ProQuest Historical Newspapers: The Christian Science Monitor, ProQuest Historical Newspapers: The Cincinnati Enquirer, ProQuest Historical Newspapers: The Globe and Mail, ProQuest Historical Newspapers: The Globe and Mail, ProQuest Historical Newspapers: The Guardian and The Observer, ProQuest Historical Newspapers: The Guardian and The Observer, ProQuest Historical Newspapers: The Irish Times and The Weekly Irish Times, ProQuest Historical Newspapers: The Irish Times and The Weekly Irish Times, ProQuest Historical Newspapers: The Jerusalem Post, ProQuest Historical Newspapers: The Jerusalem Post, ProQuest Historical Newspapers: The Jewish Advocate, ProQuest Historical Newspapers: The Jewish Advocate, ProQuest Historical Newspapers: The Jewish Exponent, ProQuest Historical Newspapers: The Jewish Exponent, ProQuest Historical Newspapers: The Korea Times, ProQuest Historical Newspapers: The Korea Times, ProQuest Historical Newspapers: The Nashville Tennessean, ProQuest Historical Newspapers: The New York Times with Index, ProQuest Historical Newspapers: The New York Times with Index, ProQuest Historical Newspapers: The Philadelphia Inquirer, ProQuest Historical Newspapers: The Philadelphia Inquirer, ProQuest Historical Newspapers: The Province, ProQuest Historical Newspapers: The Province, ProQuest Historical Newspapers: The Scotsman, ProQuest Historical Newspapers: The Scotsman, ProQuest Historical Newspapers: The Times of India, ProQuest Historical Newspapers: The Times of India, ProQuest Historical Newspapers: The Wall Street Journal, ProQuest Historical Newspapers: The Wall Street Journal, ProQuest Historical Newspapers: The Washington Post, ProQuest Historical Newspapers: The Washington Post, ProQuest Historical Newspapers: Times Colonist, ProQuest Historical Newspapers: Times Colonist, ProQuest Historical Newspapers: Toronto Star, ProQuest Historical Newspapers: Toronto Star, ProQuest Historical Newspapers: Vancouver Sun, ProQuest Historical Newspapers: Vancouver Sun, ProQuest Historical Newspapers: Windsor Star, ProQuest Historical Newspapers: Windsor Star, ProQuest Learning: Literature, ProQuest Learning: Literature, ProQuest One Business, ProQuest One Literature, Publicly Available Content Database, Queen Victoria's Journals, Queen Victoria's Journals, Religious Magazine Archive, Religious Magazine Archive, Schillers Werke, SciTech Premium Collection, SIRS Discoverer, SIRS Issues Researcher, Socialist and Radical Periodicals, Socialist and Radical Periodicals, Social Science Premium Collection, Sports Medicine & Education Index, Teatro Español del Siglo de Oro, The Annual Register: A Record of World Events, The Annual Register: A Record of World Events, The Artforum Archive, The Artforum Archive, The Cecil Papers, The Cecil Papers, The Far Eastern Economic Review Archive, The Far Eastern Economic Review Archive, The GQ Archive, The GQ Archive, The Harper's Bazaar Archive, The Harper's Bazaar Archive, The House Beautiful Archive, The House Beautiful Archive, The Newsweek Archive, The Newsweek Archive, The Rolling Stone Archive, The Rolling Stone Archive, The Vogue Archive, The Vogue Archive, The Vogue Italia Archive, The Vogue Italia Archive, The Women's Wear Daily Archive, The Women's Wear Daily Archive, Trench Journals and Unit Magazines of the First World War, Trench Journals and Unit Magazines of the First World War, Trends & Policy: U.S. Healthcare News, Trends & Policy: U.S. Immigration News, Trends & Policy: U.S. Immigration News, Twentieth-Century African American Poetry, Twentieth-Century African American Poetry, Twentieth-Century American Poetry, Twentieth-Century American Poetry, Twentieth-Century American Poetry, Second Edition, Twentieth-Century Drama, Women's Magazine Archive, Youth and Popular Culture Magazine Archive, Youth and Popular Culture Magazine Archive

Results: 86497

Set#: S2

Searched for: AB,TI(''Continuous renal replacement therapy'' OR ''Continuous RRT'' OR ''Continuous Renal Replacement Procedure'' OR ''CRRT'' OR ''Continuous Venovenous Hemofiltration'' OR ''CVVH'' OR ''Continuous Veno Venous Hemodialysis'' OR ''CVVHD'' OR ''Continuous Veno Venous Hemodiafiltration'' OR ''CVVHDF'' OR ''Continuous blood purification'' OR ''CBP'' OR ''Extracorporeal Blood Purification'')

Databases: Acta Sanctorum, Acta Sanctorum, African American Poetry, African Writers Series, African Writers Series, Alt-PressWatch, American Periodicals, American Poetry, Annual Bibliography of English Language and Literature (ABELL), Archive Finder, Art & Architecture Archive, Arts Premium Collection, Bertolt Brechts Werke, Bibliografía de la Literatura Española, Black Abolitionist Papers, Black Abolitionist Papers, Black Studies Center, British Periodicals, C19: The Nineteenth Century Index, Canadian Poetry, Children's Magazine Archive, 1866-2020, Children's Magazine Archive, 1866-2020, Colonial Legacies: Empire & Commonwealth Periodicals, Colonial Legacies: Empire & Commonwealth Periodicals, Colonial State Papers, Colonial State Papers, Coronavirus Research Database, Country Life Archive, Country Life Archive, Die Deutsche Lyrik in Reclams Universal-Bibliothek, Digital National Security Archive, Documents on British Policy Overseas, Documents on British Policy Overseas, Early American Fiction 1789-1875, Early English Prose Fiction, Early Modern Books, Ebook Central, Economist Intelligence Unit Country Reports Archive, Economist Intelligence Unit Country Reports Archive, Education Magazine Archive, Education Magazine Archive, Eighteenth-Century Fiction, eLibrary, English Drama, English Poetry, Second Edition, Entertainment Industry Magazine Archive, Ethnic NewsWatch, GenderWatch, GeoRef, Gerritsen Women's History Collection of Aletta H. Jacobs, Gerritsen Women's History Collection of Aletta H. Jacobs, Goethes Werke, Health & Fitness Magazine Archive, Health & Fitness Magazine Archive, Health Research Premium Collection, Historic Literary Criticism, History Study Center, History Study Center, Humanities Index, John Johnson Collection: An Archive of Printed Ephemera, John Johnson Collection: An Archive of Printed Ephemera, Kafkas Werke, Latinx Thought and Culture: The NPR Archive, 1979-1990, Latinx Thought and Culture: The NPR Archive, 1979-1990, LGBT Magazine Archive, LGBT Magazine Archive Collection 1, Literature Online, Men’s Magazine Archive, Men’s Magazine Archive, News, Policy & Politics Magazine Archive (feat. Newsweek), News, Policy & Politics Magazine Archive (feat. Newsweek), Nineteenth-Century Fiction, Patrologia Latina, Patrologia Latina, Periodicals Archive Online, Periodicals Index Online, Philosopher's Index, Policy File Index, PRISMA Database, PRISMA Database, ProQuest Central, ProQuest Civil War Era, ProQuest Civil War Era, ProQuest Dissertations & Theses Global, ProQuest Historical Annual Reports, ProQuest Historical Newspapers: Atlanta Daily World, ProQuest Historical Newspapers: Atlanta Daily World, ProQuest Historical Newspapers: Calgary Herald, ProQuest Historical Newspapers: Calgary Herald, ProQuest Historical Newspapers: Chicago Defender, ProQuest Historical Newspapers: Chicago Defender, ProQuest Historical Newspapers: Chicago Defender, ProQuest Historical Newspapers: Chicago Defender, ProQuest Historical Newspapers: Chicago Tribune, ProQuest Historical Newspapers: Chicago Tribune, ProQuest Historical Newspapers: Chicago Tribune, ProQuest Historical Newspapers: Chinese Newspapers Collection, ProQuest Historical Newspapers: Chinese Newspapers Collection, ProQuest Historical Newspapers: Cleveland Call and Post, ProQuest Historical Newspapers: Cleveland Call and Post, ProQuest Historical Newspapers: Communist Historical Newspaper Collection, ProQuest Historical Newspapers: Communist Historical Newspaper Collection, ProQuest Historical Newspapers: Dayton Daily News, ProQuest Historical Newspapers: Dayton Daily News, ProQuest Historical Newspapers: Detroit Free Press, ProQuest Historical Newspapers: Detroit Free Press (1831-1922), ProQuest Historical Newspapers: Edmonton Journal, ProQuest Historical Newspapers: Edmonton Journal, ProQuest Historical Newspapers: Hartford Courant, ProQuest Historical Newspapers: Hartford Courant, ProQuest Historical Newspapers: Indianapolis Star, ProQuest Historical Newspapers: Indianapolis Star (1903-1922), ProQuest Historical Newspapers: Leader-Post, ProQuest Historical Newspapers: Leader-Post, ProQuest Historical Newspapers: Leftist Newspapers and Periodicals, ProQuest Historical Newspapers: Leftist Newspapers and Periodicals, ProQuest Historical Newspapers: Los Angeles Sentinel, ProQuest Historical Newspapers: Los Angeles Sentinel, ProQuest Historical Newspapers: Los Angeles Times, ProQuest Historical Newspapers: Los Angeles Times, ProQuest Historical Newspapers: Los Angeles Times, ProQuest Historical Newspapers: Louisville Courier Journal, ProQuest Historical Newspapers: Michigan Chronicle, ProQuest Historical Newspapers: Michigan Chronicle, ProQuest Historical Newspapers: Minneapolis Star Tribune, ProQuest Historical Newspapers: Minneapolis Star Tribune, ProQuest Historical Newspapers: Montreal Gazette, ProQuest Historical Newspapers: Montreal Gazette, ProQuest Historical Newspapers: Newsday, ProQuest Historical Newspapers: Newsday, ProQuest Historical Newspapers: New York Amsterdam News, ProQuest Historical Newspapers: New York Tribune, ProQuest Historical Newspapers: Norfolk Journal and Guide, ProQuest Historical Newspapers: Norfolk Journal and Guide, ProQuest Historical Newspapers: Ottawa Citizen, ProQuest Historical Newspapers: Ottawa Citizen, ProQuest Historical Newspapers: Philadelphia Tribune, ProQuest Historical Newspapers: Philadelphia Tribune, ProQuest Historical Newspapers: Pittsburgh Courier, ProQuest Historical Newspapers: Pittsburgh Courier, ProQuest Historical Newspapers: Pittsburgh Post-Gazette, ProQuest Historical Newspapers: Pittsburgh Post-Gazette, ProQuest Historical Newspapers: San Francisco Chronicle, ProQuest Historical Newspapers: San Francisco Chronicle, ProQuest Historical Newspapers: Saskatoon Star-Phoenix, ProQuest Historical Newspapers: Saskatoon Star-Phoenix, ProQuest Historical Newspapers: South China Morning Post, ProQuest Historical Newspapers: South China Morning Post, ProQuest Historical Newspapers: St. Louis Post Dispatch, ProQuest Historical Newspapers: St. Louis Post-Dispatch (1874-1922), ProQuest Historical Newspapers: St. Petersburg Times / Tampa Bay Times, ProQuest Historical Newspapers: St. Petersburg Times / Tampa Bay Times, ProQuest Historical Newspapers: The American Hebrew & Jewish Messenger, ProQuest Historical Newspapers: The American Hebrew & Jewish Messenger, ProQuest Historical Newspapers: The American Israelite, ProQuest Historical Newspapers: The American Israelite, ProQuest Historical Newspapers: The Arizona Republican, ProQuest Historical Newspapers: The Arizona Republican (1890-1922), ProQuest Historical Newspapers: The Atlanta Constitution, ProQuest Historical Newspapers: The Atlanta Constitution, ProQuest Historical Newspapers: The Atlanta Constitution, ProQuest Historical Newspapers: The Baltimore Afro-American, ProQuest Historical Newspapers: The Baltimore Afro-American, ProQuest Historical Newspapers: The Baltimore Sun, ProQuest Historical Newspapers: The Baltimore Sun, ProQuest Historical Newspapers: The Boston Globe, ProQuest Historical Newspapers: The Boston Globe, ProQuest Historical Newspapers: The Christian Science Monitor, ProQuest Historical Newspapers: The Christian Science Monitor, ProQuest Historical Newspapers: The Cincinnati Enquirer, ProQuest Historical Newspapers: The Globe and Mail, ProQuest Historical Newspapers: The Globe and Mail, ProQuest Historical Newspapers: The Guardian and The Observer, ProQuest Historical Newspapers: The Guardian and The Observer, ProQuest Historical Newspapers: The Irish Times and The Weekly Irish Times, ProQuest Historical Newspapers: The Irish Times and The Weekly Irish Times, ProQuest Historical Newspapers: The Jerusalem Post, ProQuest Historical Newspapers: The Jerusalem Post, ProQuest Historical Newspapers: The Jewish Advocate, ProQuest Historical Newspapers: The Jewish Advocate, ProQuest Historical Newspapers: The Jewish Exponent, ProQuest Historical Newspapers: The Jewish Exponent, ProQuest Historical Newspapers: The Korea Times, ProQuest Historical Newspapers: The Korea Times, ProQuest Historical Newspapers: The Nashville Tennessean, ProQuest Historical Newspapers: The New York Times with Index, ProQuest Historical Newspapers: The New York Times with Index, ProQuest Historical Newspapers: The Philadelphia Inquirer, ProQuest Historical Newspapers: The Philadelphia Inquirer, ProQuest Historical Newspapers: The Province, ProQuest Historical Newspapers: The Province, ProQuest Historical Newspapers: The Scotsman, ProQuest Historical Newspapers: The Scotsman, ProQuest Historical Newspapers: The Times of India, ProQuest Historical Newspapers: The Times of India, ProQuest Historical Newspapers: The Wall Street Journal, ProQuest Historical Newspapers: The Wall Street Journal, ProQuest Historical Newspapers: The Washington Post, ProQuest Historical Newspapers: The Washington Post, ProQuest Historical Newspapers: Times Colonist, ProQuest Historical Newspapers: Times Colonist, ProQuest Historical Newspapers: Toronto Star, ProQuest Historical Newspapers: Toronto Star, ProQuest Historical Newspapers: Vancouver Sun, ProQuest Historical Newspapers: Vancouver Sun, ProQuest Historical Newspapers: Windsor Star, ProQuest Historical Newspapers: Windsor Star, ProQuest Learning: Literature, ProQuest Learning: Literature, ProQuest One Business, ProQuest One Literature, Publicly Available Content Database, Queen Victoria's Journals, Queen Victoria's Journals, Religious Magazine Archive, Religious Magazine Archive, Schillers Werke, SciTech Premium Collection, SIRS Discoverer, SIRS Issues Researcher, Socialist and Radical Periodicals, Socialist and Radical Periodicals, Social Science Premium Collection, Sports Medicine & Education Index, Teatro Español del Siglo de Oro, The Annual Register: A Record of World Events, The Annual Register: A Record of World Events, The Artforum Archive, The Artforum Archive, The Cecil Papers, The Cecil Papers, The Far Eastern Economic Review Archive, The Far Eastern Economic Review Archive, The GQ Archive, The GQ Archive, The Harper's Bazaar Archive, The Harper's Bazaar Archive, The House Beautiful Archive, The House Beautiful Archive, The Newsweek Archive, The Newsweek Archive, The Rolling Stone Archive, The Rolling Stone Archive, The Vogue Archive, The Vogue Archive, The Vogue Italia Archive, The Vogue Italia Archive, The Women's Wear Daily Archive, The Women's Wear Daily Archive, Trench Journals and Unit Magazines of the First World War, Trench Journals and Unit Magazines of the First World War, Trends & Policy: U.S. Healthcare News, Trends & Policy: U.S. Immigration News, Trends & Policy: U.S. Immigration News, Twentieth-Century African American Poetry, Twentieth-Century African American Poetry, Twentieth-Century American Poetry, Twentieth-Century American Poetry, Twentieth-Century American Poetry, Second Edition, Twentieth-Century Drama, Women's Magazine Archive, Youth and Popular Culture Magazine Archive, Youth and Popular Culture Magazine Archive

Results: 55164

Set#: S3

Searched for: AB,TI(''randomized controlled trial'' OR ''randomized'' OR ''placebo'')

Databases: Acta Sanctorum, Acta Sanctorum, African American Poetry, African Writers Series, African Writers Series, Alt-PressWatch, American Periodicals, American Poetry, Annual Bibliography of English Language and Literature (ABELL), Archive Finder, Art & Architecture Archive, Arts Premium Collection, Bertolt Brechts Werke, Bibliografía de la Literatura Española, Black Abolitionist Papers, Black Abolitionist Papers, Black Studies Center, British Periodicals, C19: The Nineteenth Century Index, Canadian Poetry, Children's Magazine Archive, 1866-2020, Children's Magazine Archive, 1866-2020, Colonial Legacies: Empire & Commonwealth Periodicals, Colonial Legacies: Empire & Commonwealth Periodicals, Colonial State Papers, Colonial State Papers, Coronavirus Research Database, Country Life Archive, Country Life Archive, Die Deutsche Lyrik in Reclams Universal-Bibliothek, Digital National Security Archive, Documents on British Policy Overseas, Documents on British Policy Overseas, Early American Fiction 1789-1875, Early English Prose Fiction, Early Modern Books, Ebook Central, Economist Intelligence Unit Country Reports Archive, Economist Intelligence Unit Country Reports Archive, Education Magazine Archive, Education Magazine Archive, Eighteenth-Century Fiction, eLibrary, English Drama, English Poetry, Second Edition, Entertainment Industry Magazine Archive, Ethnic NewsWatch, GenderWatch, GeoRef, Gerritsen Women's History Collection of Aletta H. Jacobs, Gerritsen Women's History Collection of Aletta H. Jacobs, Goethes Werke, Health & Fitness Magazine Archive, Health & Fitness Magazine Archive, Health Research Premium Collection, Historic Literary Criticism, History Study Center, History Study Center, Humanities Index, John Johnson Collection: An Archive of Printed Ephemera, John Johnson Collection: An Archive of Printed Ephemera, Kafkas Werke, Latinx Thought and Culture: The NPR Archive, 1979-1990, Latinx Thought and Culture: The NPR Archive, 1979-1990, LGBT Magazine Archive, LGBT Magazine Archive Collection 1, Literature Online, Men’s Magazine Archive, Men’s Magazine Archive, News, Policy & Politics Magazine Archive (feat. Newsweek), News, Policy & Politics Magazine Archive (feat. Newsweek), Nineteenth-Century Fiction, Patrologia Latina, Patrologia Latina, Periodicals Archive Online, Periodicals Index Online, Philosopher's Index, Policy File Index, PRISMA Database, PRISMA Database, ProQuest Central, ProQuest Civil War Era, ProQuest Civil War Era, ProQuest Dissertations & Theses Global, ProQuest Historical Annual Reports, ProQuest Historical Newspapers: Atlanta Daily World, ProQuest Historical Newspapers: Atlanta Daily World, ProQuest Historical Newspapers: Calgary Herald, ProQuest Historical Newspapers: Calgary Herald, ProQuest Historical Newspapers: Chicago Defender, ProQuest Historical Newspapers: Chicago Defender, ProQuest Historical Newspapers: Chicago Defender, ProQuest Historical Newspapers: Chicago Defender, ProQuest Historical Newspapers: Chicago Tribune, ProQuest Historical Newspapers: Chicago Tribune, ProQuest Historical Newspapers: Chicago Tribune, ProQuest Historical Newspapers: Chinese Newspapers Collection, ProQuest Historical Newspapers: Chinese Newspapers Collection, ProQuest Historical Newspapers: Cleveland Call and Post, ProQuest Historical Newspapers: Cleveland Call and Post, ProQuest Historical Newspapers: Communist Historical Newspaper Collection, ProQuest Historical Newspapers: Communist Historical Newspaper Collection, ProQuest Historical Newspapers: Dayton Daily News, ProQuest Historical Newspapers: Dayton Daily News, ProQuest Historical Newspapers: Detroit Free Press, ProQuest Historical Newspapers: Detroit Free Press (1831-1922), ProQuest Historical Newspapers: Edmonton Journal, ProQuest Historical Newspapers: Edmonton Journal, ProQuest Historical Newspapers: Hartford Courant, ProQuest Historical Newspapers: Hartford Courant, ProQuest Historical Newspapers: Indianapolis Star, ProQuest Historical Newspapers: Indianapolis Star (1903-1922), ProQuest Historical Newspapers: Leader-Post, ProQuest Historical Newspapers: Leader-Post, ProQuest Historical Newspapers: Leftist Newspapers and Periodicals, ProQuest Historical Newspapers: Leftist Newspapers and Periodicals, ProQuest Historical Newspapers: Los Angeles Sentinel, ProQuest Historical Newspapers: Los Angeles Sentinel, ProQuest Historical Newspapers: Los Angeles Times, ProQuest Historical Newspapers: Los Angeles Times, ProQuest Historical Newspapers: Los Angeles Times, ProQuest Historical Newspapers: Louisville Courier Journal, ProQuest Historical Newspapers: Michigan Chronicle, ProQuest Historical Newspapers: Michigan Chronicle, ProQuest Historical Newspapers: Minneapolis Star Tribune, ProQuest Historical Newspapers: Minneapolis Star Tribune, ProQuest Historical Newspapers: Montreal Gazette, ProQuest Historical Newspapers: Montreal Gazette, ProQuest Historical Newspapers: Newsday, ProQuest Historical Newspapers: Newsday, ProQuest Historical Newspapers: New York Amsterdam News, ProQuest Historical Newspapers: New York Tribune, ProQuest Historical Newspapers: Norfolk Journal and Guide, ProQuest Historical Newspapers: Norfolk Journal and Guide, ProQuest Historical Newspapers: Ottawa Citizen, ProQuest Historical Newspapers: Ottawa Citizen, ProQuest Historical Newspapers: Philadelphia Tribune, ProQuest Historical Newspapers: Philadelphia Tribune, ProQuest Historical Newspapers: Pittsburgh Courier, ProQuest Historical Newspapers: Pittsburgh Courier, ProQuest Historical Newspapers: Pittsburgh Post-Gazette, ProQuest Historical Newspapers: Pittsburgh Post-Gazette, ProQuest Historical Newspapers: San Francisco Chronicle, ProQuest Historical Newspapers: San Francisco Chronicle, ProQuest Historical Newspapers: Saskatoon Star-Phoenix, ProQuest Historical Newspapers: Saskatoon Star-Phoenix, ProQuest Historical Newspapers: South China Morning Post, ProQuest Historical Newspapers: South China Morning Post, ProQuest Historical Newspapers: St. Louis Post Dispatch, ProQuest Historical Newspapers: St. Louis Post-Dispatch (1874-1922), ProQuest Historical Newspapers: St. Petersburg Times / Tampa Bay Times, ProQuest Historical Newspapers: St. Petersburg Times / Tampa Bay Times, ProQuest Historical Newspapers: The American Hebrew & Jewish Messenger, ProQuest Historical Newspapers: The American Hebrew & Jewish Messenger, ProQuest Historical Newspapers: The American Israelite, ProQuest Historical Newspapers: The American Israelite, ProQuest Historical Newspapers: The Arizona Republican, ProQuest Historical Newspapers: The Arizona Republican (1890-1922), ProQuest Historical Newspapers: The Atlanta Constitution, ProQuest Historical Newspapers: The Atlanta Constitution, ProQuest Historical Newspapers: The Atlanta Constitution, ProQuest Historical Newspapers: The Baltimore Afro-American, ProQuest Historical Newspapers: The Baltimore Afro-American, ProQuest Historical Newspapers: The Baltimore Sun, ProQuest Historical Newspapers: The Baltimore Sun, ProQuest Historical Newspapers: The Boston Globe, ProQuest Historical Newspapers: The Boston Globe, ProQuest Historical Newspapers: The Christian Science Monitor, ProQuest Historical Newspapers: The Christian Science Monitor, ProQuest Historical Newspapers: The Cincinnati Enquirer, ProQuest Historical Newspapers: The Globe and Mail, ProQuest Historical Newspapers: The Globe and Mail, ProQuest Historical Newspapers: The Guardian and The Observer, ProQuest Historical Newspapers: The Guardian and The Observer, ProQuest Historical Newspapers: The Irish Times and The Weekly Irish Times, ProQuest Historical Newspapers: The Irish Times and The Weekly Irish Times, ProQuest Historical Newspapers: The Jerusalem Post, ProQuest Historical Newspapers: The Jerusalem Post, ProQuest Historical Newspapers: The Jewish Advocate, ProQuest Historical Newspapers: The Jewish Advocate, ProQuest Historical Newspapers: The Jewish Exponent, ProQuest Historical Newspapers: The Jewish Exponent, ProQuest Historical Newspapers: The Korea Times, ProQuest Historical Newspapers: The Korea Times, ProQuest Historical Newspapers: The Nashville Tennessean, ProQuest Historical Newspapers: The New York Times with Index, ProQuest Historical Newspapers: The New York Times with Index, ProQuest Historical Newspapers: The Philadelphia Inquirer, ProQuest Historical Newspapers: The Philadelphia Inquirer, ProQuest Historical Newspapers: The Province, ProQuest Historical Newspapers: The Province, ProQuest Historical Newspapers: The Scotsman, ProQuest Historical Newspapers: The Scotsman, ProQuest Historical Newspapers: The Times of India, ProQuest Historical Newspapers: The Times of India, ProQuest Historical Newspapers: The Wall Street Journal, ProQuest Historical Newspapers: The Wall Street Journal, ProQuest Historical Newspapers: The Washington Post, ProQuest Historical Newspapers: The Washington Post, ProQuest Historical Newspapers: Times Colonist, ProQuest Historical Newspapers: Times Colonist, ProQuest Historical Newspapers: Toronto Star, ProQuest Historical Newspapers: Toronto Star, ProQuest Historical Newspapers: Vancouver Sun, ProQuest Historical Newspapers: Vancouver Sun, ProQuest Historical Newspapers: Windsor Star, ProQuest Historical Newspapers: Windsor Star, ProQuest Learning: Literature, ProQuest Learning: Literature, ProQuest One Business, ProQuest One Literature, Publicly Available Content Database, Queen Victoria's Journals, Queen Victoria's Journals, Religious Magazine Archive, Religious Magazine Archive, Schillers Werke, SciTech Premium Collection, SIRS Discoverer, SIRS Issues Researcher, Socialist and Radical Periodicals, Socialist and Radical Periodicals, Social Science Premium Collection, Sports Medicine & Education Index, Teatro Español del Siglo de Oro, The Annual Register: A Record of World Events, The Annual Register: A Record of World Events, The Artforum Archive, The Artforum Archive, The Cecil Papers, The Cecil Papers, The Far Eastern Economic Review Archive, The Far Eastern Economic Review Archive, The GQ Archive, The GQ Archive, The Harper's Bazaar Archive, The Harper's Bazaar Archive, The House Beautiful Archive, The House Beautiful Archive, The Newsweek Archive, The Newsweek Archive, The Rolling Stone Archive, The Rolling Stone Archive, The Vogue Archive, The Vogue Archive, The Vogue Italia Archive, The Vogue Italia Archive, The Women's Wear Daily Archive, The Women's Wear Daily Archive, Trench Journals and Unit Magazines of the First World War, Trench Journals and Unit Magazines of the First World War, Trends & Policy: U.S. Healthcare News, Trends & Policy: U.S. Immigration News, Trends & Policy: U.S. Immigration News, Twentieth-Century African American Poetry, Twentieth-Century African American Poetry, Twentieth-Century American Poetry, Twentieth-Century American Poetry, Twentieth-Century American Poetry, Second Edition, Twentieth-Century Drama, Women's Magazine Archive, Youth and Popular Culture Magazine Archive, Youth and Popular Culture Magazine Archive

Results: 1671661

Set#: S4

Searched for: [S1] AND [S2] AND [S3]

Databases: Acta Sanctorum, Acta Sanctorum, African American Poetry, African Writers Series, African Writers Series, Alt-PressWatch, American Periodicals, American Poetry, Annual Bibliography of English Language and Literature (ABELL), Archive Finder, Art & Architecture Archive, Arts Premium Collection, Bertolt Brechts Werke, Bibliografía de la Literatura Española, Black Abolitionist Papers, Black Abolitionist Papers, Black Studies Center, British Periodicals, C19: The Nineteenth Century Index, Canadian Poetry, Children's Magazine Archive, 1866-2020, Children's Magazine Archive, 1866-2020, Colonial Legacies: Empire & Commonwealth Periodicals, Colonial Legacies: Empire & Commonwealth Periodicals, Colonial State Papers, Colonial State Papers, Coronavirus Research Database, Country Life Archive, Country Life Archive, Die Deutsche Lyrik in Reclams Universal-Bibliothek, Digital National Security Archive, Documents on British Policy Overseas, Documents on British Policy Overseas, Early American Fiction 1789-1875, Early English Prose Fiction, Early Modern Books, Ebook Central, Economist Intelligence Unit Country Reports Archive, Economist Intelligence Unit Country Reports Archive, Education Magazine Archive, Education Magazine Archive, Eighteenth-Century Fiction, eLibrary, English Drama, English Poetry, Second Edition, Entertainment Industry Magazine Archive, Ethnic NewsWatch, GenderWatch, GeoRef, Gerritsen Women's History Collection of Aletta H. Jacobs, Gerritsen Women's History Collection of Aletta H. Jacobs, Goethes Werke, Health & Fitness Magazine Archive, Health & Fitness Magazine Archive, Health Research Premium Collection, Historic Literary Criticism, History Study Center, History Study Center, Humanities Index, John Johnson Collection: An Archive of Printed Ephemera, John Johnson Collection: An Archive of Printed Ephemera, Kafkas Werke, Latinx Thought and Culture: The NPR Archive, 1979-1990, Latinx Thought and Culture: The NPR Archive, 1979-1990, LGBT Magazine Archive, LGBT Magazine Archive Collection 1, Literature Online, Men’s Magazine Archive, Men’s Magazine Archive, News, Policy & Politics Magazine Archive (feat. Newsweek), News, Policy & Politics Magazine Archive (feat. Newsweek), Nineteenth-Century Fiction, Patrologia Latina, Patrologia Latina, Periodicals Archive Online, Periodicals Index Online, Philosopher's Index, Policy File Index, PRISMA Database, PRISMA Database, ProQuest Central, ProQuest Civil War Era, ProQuest Civil War Era, ProQuest Dissertations & Theses Global, ProQuest Historical Annual Reports, ProQuest Historical Newspapers: Atlanta Daily World, ProQuest Historical Newspapers: Atlanta Daily World, ProQuest Historical Newspapers: Calgary Herald, ProQuest Historical Newspapers: Calgary Herald, ProQuest Historical Newspapers: Chicago Defender, ProQuest Historical Newspapers: Chicago Defender, ProQuest Historical Newspapers: Chicago Defender, ProQuest Historical Newspapers: Chicago Defender, ProQuest Historical Newspapers: Chicago Tribune, ProQuest Historical Newspapers: Chicago Tribune, ProQuest Historical Newspapers: Chicago Tribune, ProQuest Historical Newspapers: Chinese Newspapers Collection, ProQuest Historical Newspapers: Chinese Newspapers Collection, ProQuest Historical Newspapers: Cleveland Call and Post, ProQuest Historical Newspapers: Cleveland Call and Post, ProQuest Historical Newspapers: Communist Historical Newspaper Collection, ProQuest Historical Newspapers: Communist Historical Newspaper Collection, ProQuest Historical Newspapers: Dayton Daily News, ProQuest Historical Newspapers: Dayton Daily News, ProQuest Historical Newspapers: Detroit Free Press, ProQuest Historical Newspapers: Detroit Free Press (1831-1922), ProQuest Historical Newspapers: Edmonton Journal, ProQuest Historical Newspapers: Edmonton Journal, ProQuest Historical Newspapers: Hartford Courant, ProQuest Historical Newspapers: Hartford Courant, ProQuest Historical Newspapers: Indianapolis Star, ProQuest Historical Newspapers: Indianapolis Star (1903-1922), ProQuest Historical Newspapers: Leader-Post, ProQuest Historical Newspapers: Leader-Post, ProQuest Historical Newspapers: Leftist Newspapers and Periodicals, ProQuest Historical Newspapers: Leftist Newspapers and Periodicals, ProQuest Historical Newspapers: Los Angeles Sentinel, ProQuest Historical Newspapers: Los Angeles Sentinel, ProQuest Historical Newspapers: Los Angeles Times, ProQuest Historical Newspapers: Los Angeles Times, ProQuest Historical Newspapers: Los Angeles Times, ProQuest Historical Newspapers: Louisville Courier Journal, ProQuest Historical Newspapers: Michigan Chronicle, ProQuest Historical Newspapers: Michigan Chronicle, ProQuest Historical Newspapers: Minneapolis Star Tribune, ProQuest Historical Newspapers: Minneapolis Star Tribune, ProQuest Historical Newspapers: Montreal Gazette, ProQuest Historical Newspapers: Montreal Gazette, ProQuest Historical Newspapers: Newsday, ProQuest Historical Newspapers: Newsday, ProQuest Historical Newspapers: New York Amsterdam News, ProQuest Historical Newspapers: New York Tribune, ProQuest Historical Newspapers: Norfolk Journal and Guide, ProQuest Historical Newspapers: Norfolk Journal and Guide, ProQuest Historical Newspapers: Ottawa Citizen, ProQuest Historical Newspapers: Ottawa Citizen, ProQuest Historical Newspapers: Philadelphia Tribune, ProQuest Historical Newspapers: Philadelphia Tribune, ProQuest Historical Newspapers: Pittsburgh Courier, ProQuest Historical Newspapers: Pittsburgh Courier, ProQuest Historical Newspapers: Pittsburgh Post-Gazette, ProQuest Historical Newspapers: Pittsburgh Post-Gazette, ProQuest Historical Newspapers: San Francisco Chronicle, ProQuest Historical Newspapers: San Francisco Chronicle, ProQuest Historical Newspapers: Saskatoon Star-Phoenix, ProQuest Historical Newspapers: Saskatoon Star-Phoenix, ProQuest Historical Newspapers: South China Morning Post, ProQuest Historical Newspapers: South China Morning Post, ProQuest Historical Newspapers: St. Louis Post Dispatch, ProQuest Historical Newspapers: St. Louis Post-Dispatch (1874-1922), ProQuest Historical Newspapers: St. Petersburg Times / Tampa Bay Times, ProQuest Historical Newspapers: St. Petersburg Times / Tampa Bay Times, ProQuest Historical Newspapers: The American Hebrew & Jewish Messenger, ProQuest Historical Newspapers: The American Hebrew & Jewish Messenger, ProQuest Historical Newspapers: The American Israelite, ProQuest Historical Newspapers: The American Israelite, ProQuest Historical Newspapers: The Arizona Republican, ProQuest Historical Newspapers: The Arizona Republican (1890-1922), ProQuest Historical Newspapers: The Atlanta Constitution, ProQuest Historical Newspapers: The Atlanta Constitution, ProQuest Historical Newspapers: The Atlanta Constitution, ProQuest Historical Newspapers: The Baltimore Afro-American, ProQuest Historical Newspapers: The Baltimore Afro-American, ProQuest Historical Newspapers: The Baltimore Sun, ProQuest Historical Newspapers: The Baltimore Sun, ProQuest Historical Newspapers: The Boston Globe, ProQuest Historical Newspapers: The Boston Globe, ProQuest Historical Newspapers: The Christian Science Monitor, ProQuest Historical Newspapers: The Christian Science Monitor, ProQuest Historical Newspapers: The Cincinnati Enquirer, ProQuest Historical Newspapers: The Globe and Mail, ProQuest Historical Newspapers: The Globe and Mail, ProQuest Historical Newspapers: The Guardian and The Observer, ProQuest Historical Newspapers: The Guardian and The Observer, ProQuest Historical Newspapers: The Irish Times and The Weekly Irish Times, ProQuest Historical Newspapers: The Irish Times and The Weekly Irish Times, ProQuest Historical Newspapers: The Jerusalem Post, ProQuest Historical Newspapers: The Jerusalem Post, ProQuest Historical Newspapers: The Jewish Advocate, ProQuest Historical Newspapers: The Jewish Advocate, ProQuest Historical Newspapers: The Jewish Exponent, ProQuest Historical Newspapers: The Jewish Exponent, ProQuest Historical Newspapers: The Korea Times, ProQuest Historical Newspapers: The Korea Times, ProQuest Historical Newspapers: The Nashville Tennessean, ProQuest Historical Newspapers: The New York Times with Index, ProQuest Historical Newspapers: The New York Times with Index, ProQuest Historical Newspapers: The Philadelphia Inquirer, ProQuest Historical Newspapers: The Philadelphia Inquirer, ProQuest Historical Newspapers: The Province, ProQuest Historical Newspapers: The Province, ProQuest Historical Newspapers: The Scotsman, ProQuest Historical Newspapers: The Scotsman, ProQuest Historical Newspapers: The Times of India, ProQuest Historical Newspapers: The Times of India, ProQuest Historical Newspapers: The Wall Street Journal, ProQuest Historical Newspapers: The Wall Street Journal, ProQuest Historical Newspapers: The Washington Post, ProQuest Historical Newspapers: The Washington Post, ProQuest Historical Newspapers: Times Colonist, ProQuest Historical Newspapers: Times Colonist, ProQuest Historical Newspapers: Toronto Star, ProQuest Historical Newspapers: Toronto Star, ProQuest Historical Newspapers: Vancouver Sun, ProQuest Historical Newspapers: Vancouver Sun, ProQuest Historical Newspapers: Windsor Star, ProQuest Historical Newspapers: Windsor Star, ProQuest Learning: Literature, ProQuest Learning: Literature, ProQuest One Business, ProQuest One Literature, Publicly Available Content Database, Queen Victoria's Journals, Queen Victoria's Journals, Religious Magazine Archive, Religious Magazine Archive, Schillers Werke, SciTech Premium Collection, SIRS Discoverer, SIRS Issues Researcher, Socialist and Radical Periodicals, Socialist and Radical Periodicals, Social Science Premium Collection, Sports Medicine & Education Index, Teatro Español del Siglo de Oro, The Annual Register: A Record of World Events, The Annual Register: A Record of World Events, The Artforum Archive, The Artforum Archive, The Cecil Papers, The Cecil Papers, The Far Eastern Economic Review Archive, The Far Eastern Economic Review Archive, The GQ Archive, The GQ Archive, The Harper's Bazaar Archive, The Harper's Bazaar Archive, The House Beautiful Archive, The House Beautiful Archive, The Newsweek Archive, The Newsweek Archive, The Rolling Stone Archive, The Rolling Stone Archive, The Vogue Archive, The Vogue Archive, The Vogue Italia Archive, The Vogue Italia Archive, The Women's Wear Daily Archive, The Women's Wear Daily Archive, Trench Journals and Unit Magazines of the First World War, Trench Journals and Unit Magazines of the First World War, Trends & Policy: U.S. Healthcare News, Trends & Policy: U.S. Immigration News, Trends & Policy: U.S. Immigration News, Twentieth-Century African American Poetry, Twentieth-Century African American Poetry, Twentieth-Century American Poetry, Twentieth-Century American Poetry, Twentieth-Century American Poetry, Second Edition, Twentieth-Century Drama, Women's Magazine Archive, Youth and Popular Culture Magazine Archive, Youth and Popular Culture Magazine Archive

These databases are searched for part of your query.

Results: 20

1. Chinese clinical trial Registry

疾病类型：

主题词：急性呼吸窘迫综合征

自由词：成人呼吸窘迫综合征、ARDS、急性肺损伤、休克肺

干预措施：

主题词：连续性肾脏替代治疗

自由词：连续性肾脏替代疗法、持续性肾脏替代治疗、持续性肾脏替代疗法、连续性血液净化、持续性血液净化、连续性静脉-静脉血液滤过、连续性静脉-静脉血液透析、连续性静脉-静脉血液透析滤过、CRRT、CVVH、CVVHD、CVVHDF

研究方法：

主题词：随机对照 自由词：随机分配、随机、抽签法、随机数字表

14、Clinical Trials.gov

Disease type

Subject Word：Respiratory Distress Syndrome

random word：

Respiratory Distress Syndromes

Shock Lung

Acute Respiratory Distress Syndrome

ARDS

Human ARDS

Adult Respiratory Distress Syndrom

Intervention measures:

Subject Word：Continuous renal replacement therapy

random word：

Continuous RRT

Continuous Renal Replacement Procedure

CRRT

Continuous Venovenous Hemofiltration

CVVH

Continuous Veno Venous Hemodialysis

CVVHD

Continuous Veno Venous Hemodiafiltration

CVVHDF

Continuous blood purification

CBP

Extracorporeal Blood Purification
