# Supplementary figures and images for: New Insights on Continuous Renal Replacement Therapy for Acute Respiratory Distress Syndrome: A Systematic Review and Meta‐Analysis
Source: Clin Respir J. 2025 Jan 2;19(1):e70045. doi: 10.1111/crj.70045 (PMC11695202; doi:10.1111/crj.70045)

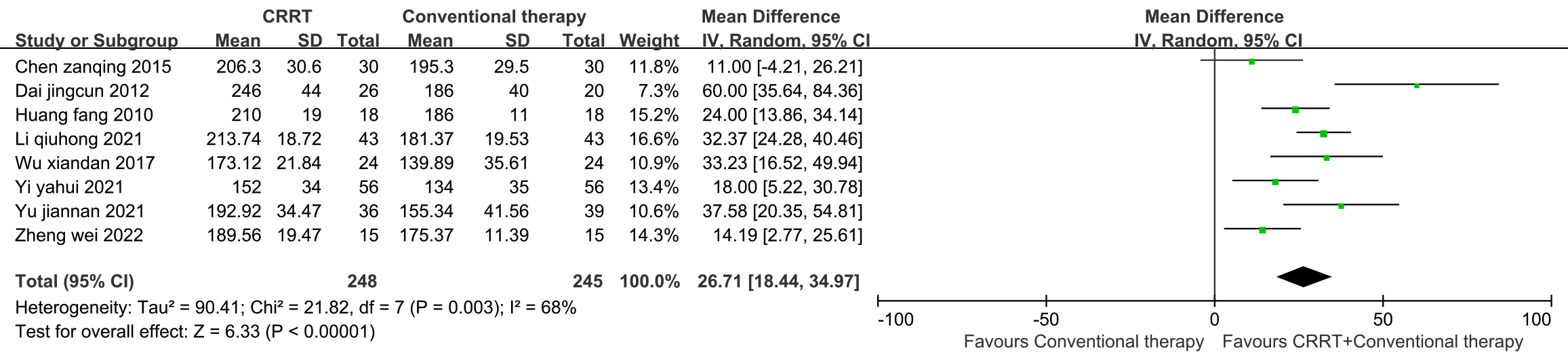

Supplement: Supplementary file 2 — Data S2 Forest‐plot of OI at 24 h. [file CRJ-19-e70045-s007.tif]

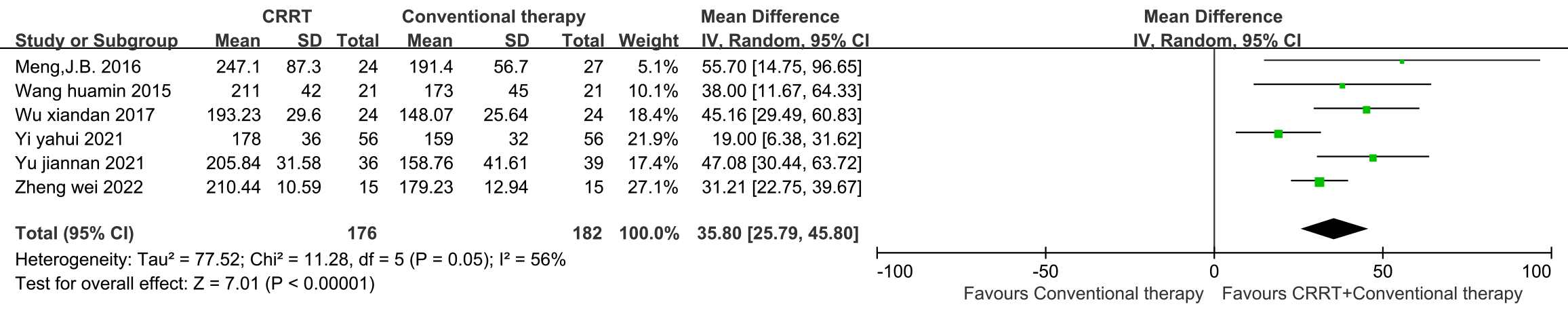

Supplement: Supplementary file 3 — Data S3 Forest‐plot of OI at 48 h. [file CRJ-19-e70045-s004.tif]

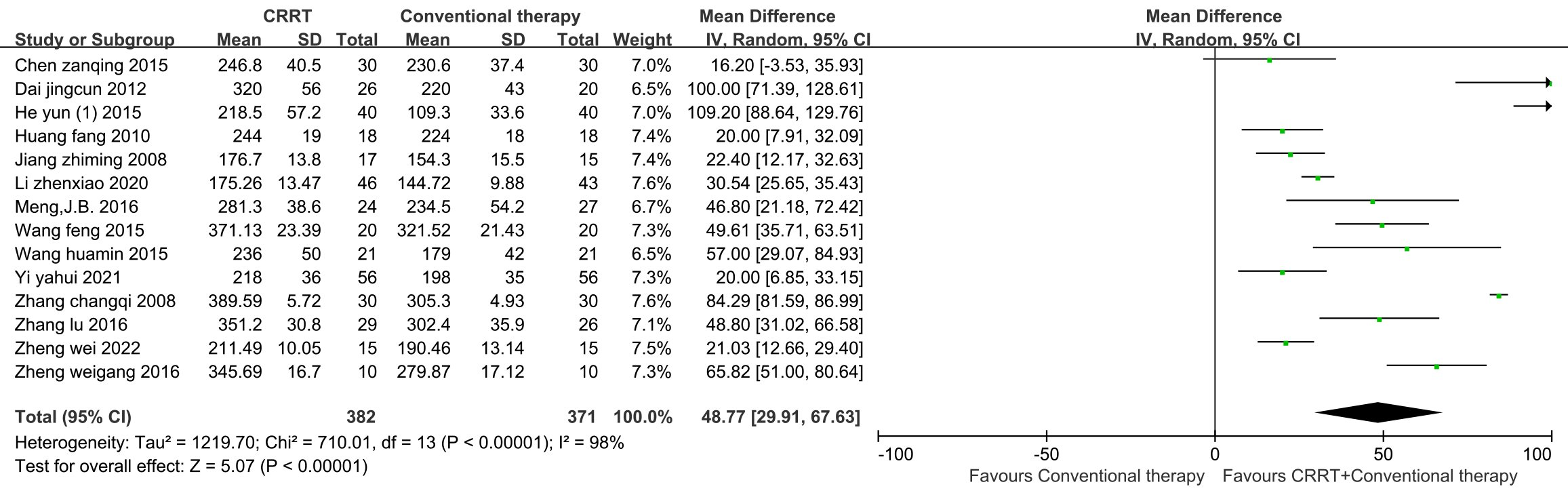

Supplement: Supplementary file 4 — Data S4 Forest‐plot of OI at 72 h. [file CRJ-19-e70045-s008.tif]

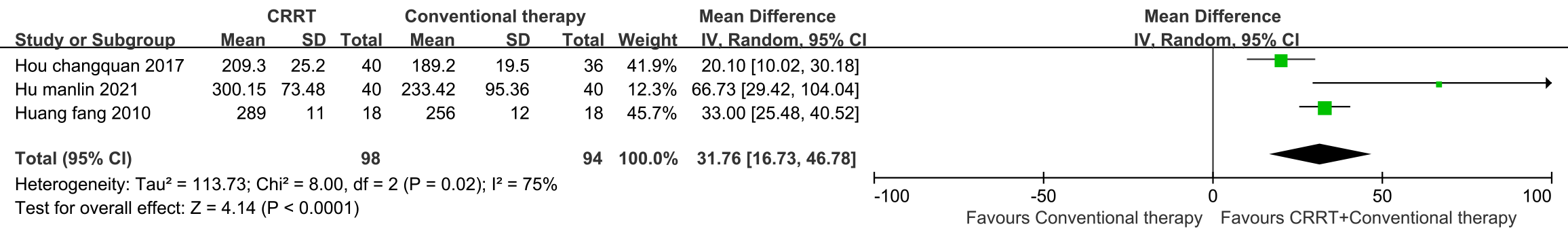

Supplement: Supplementary file 5 — Data S5 Forest‐plot of OI at 7 d. [file CRJ-19-e70045-s018.tif]

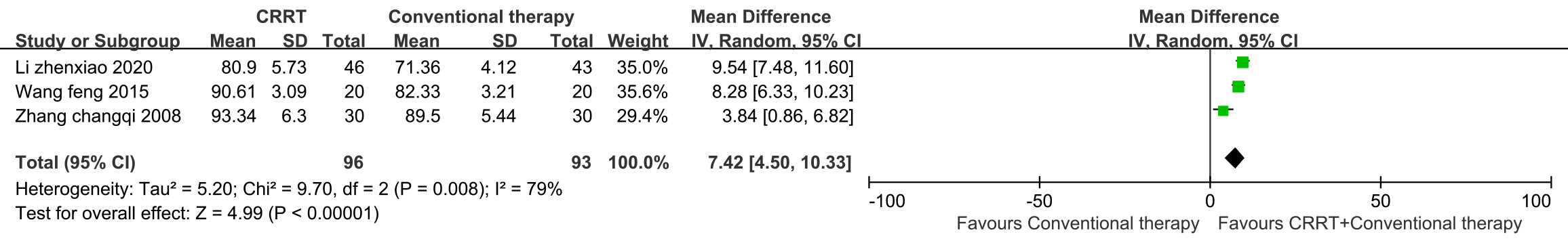

Supplement: Supplementary file 6 — Data S6 Forest‐plot of PaO2 at 72 h. [file CRJ-19-e70045-s013.tif]

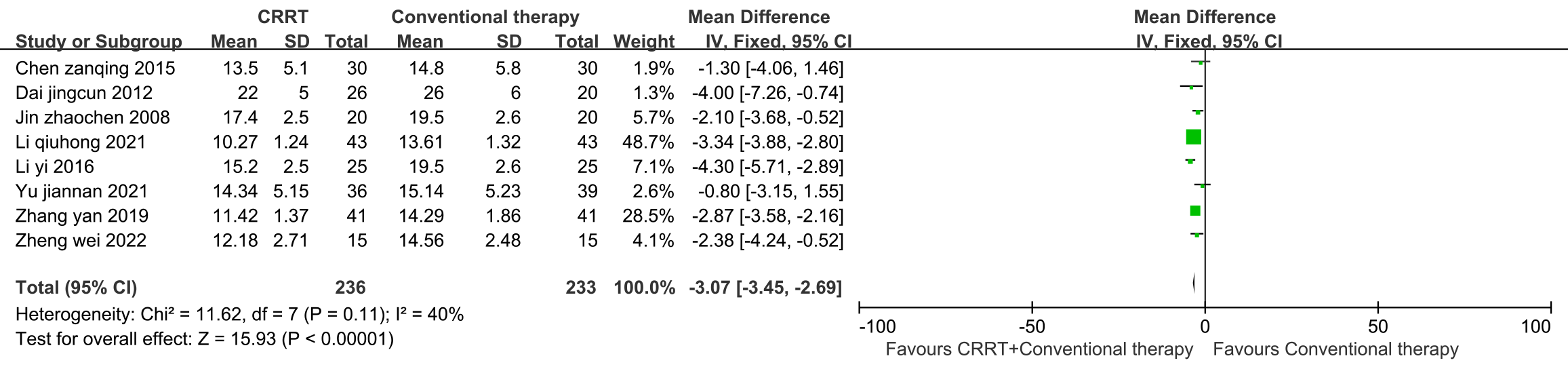

Supplement: Supplementary file 7 — Data S7 Forest‐plot of APACHE II score at 24 h. [file CRJ-19-e70045-s014.tif]

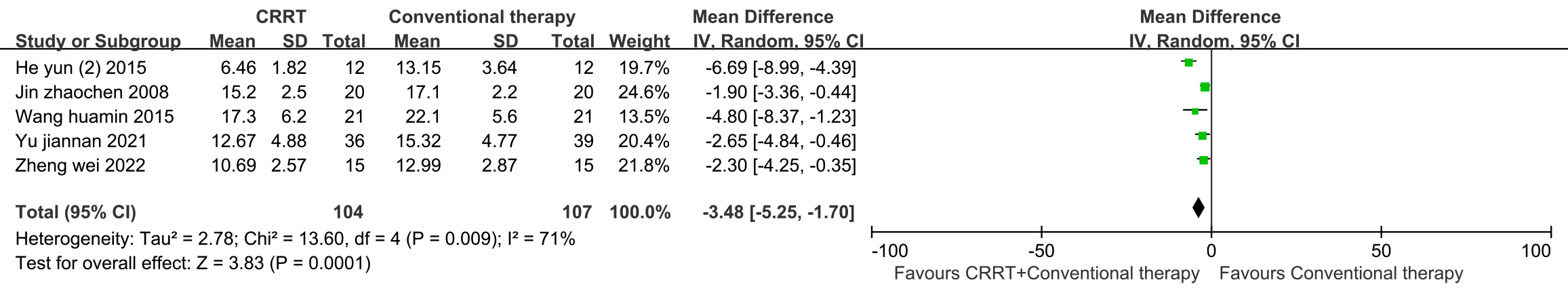

Supplement: Supplementary file 8 — Data S8 Forest‐plot of APACHE II score at 48 h. [file CRJ-19-e70045-s006.tif]

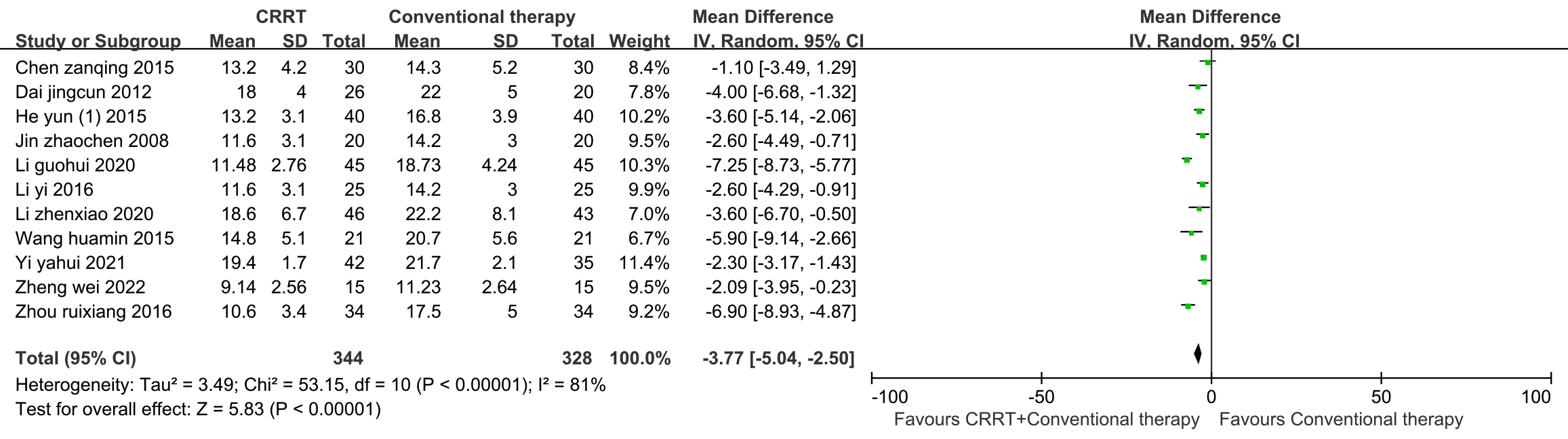

Supplement: Supplementary file 9 — Data S9 Forest‐plot of APACHE II score at 72 h. [file CRJ-19-e70045-s010.tif]

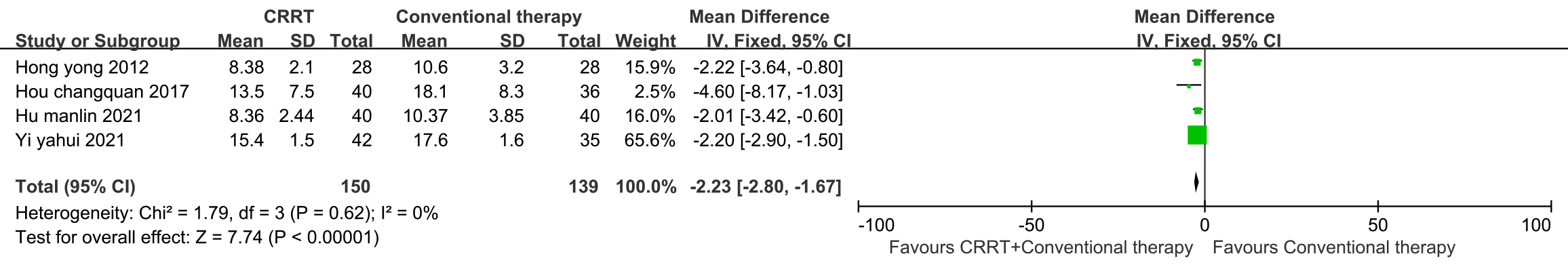

Supplement: Supplementary file 10 — Data S10 Forest‐plot of APACHE II score at 7 d. [file CRJ-19-e70045-s002.tif]

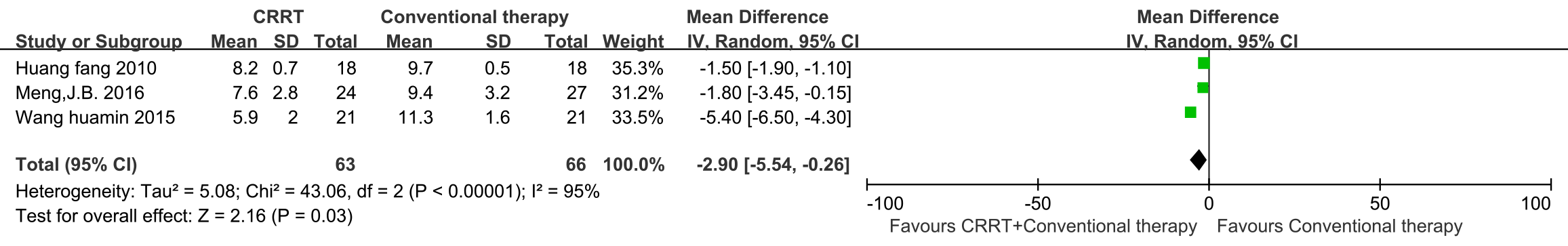

Supplement: Supplementary file 11 — Data S11 Forest‐plot of EVLWI at 72 h. [file CRJ-19-e70045-s001.tif]

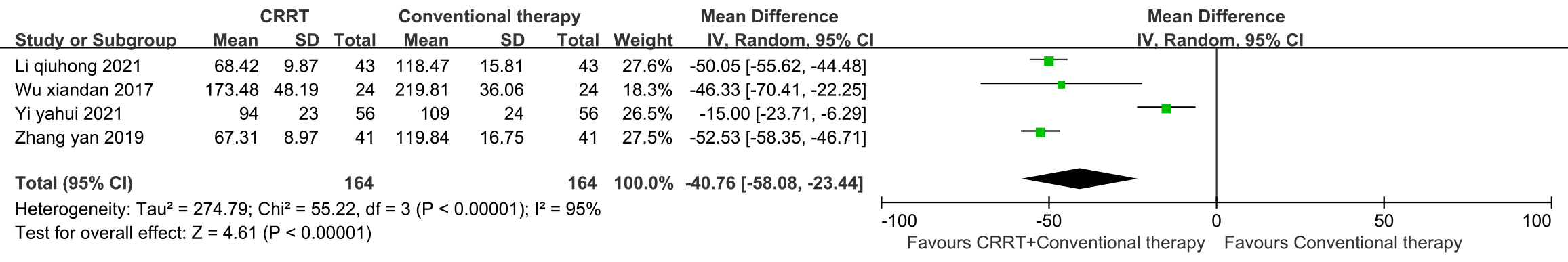

Supplement: Supplementary file 12 — Data S12 Forest‐plot of TNF‐α at 24 h. [file CRJ-19-e70045-s011.tif]

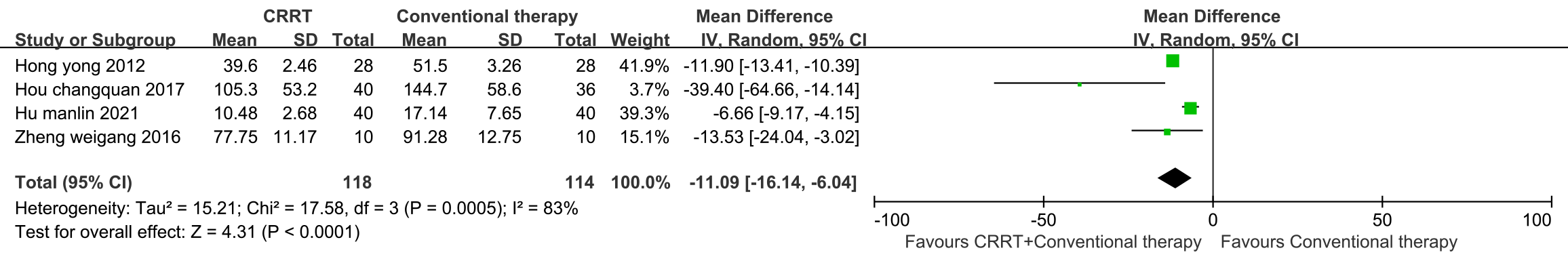

Supplement: Supplementary file 13 — Data S13 Forest‐plot of TNF‐α at 7 d. [file CRJ-19-e70045-s003.tif]

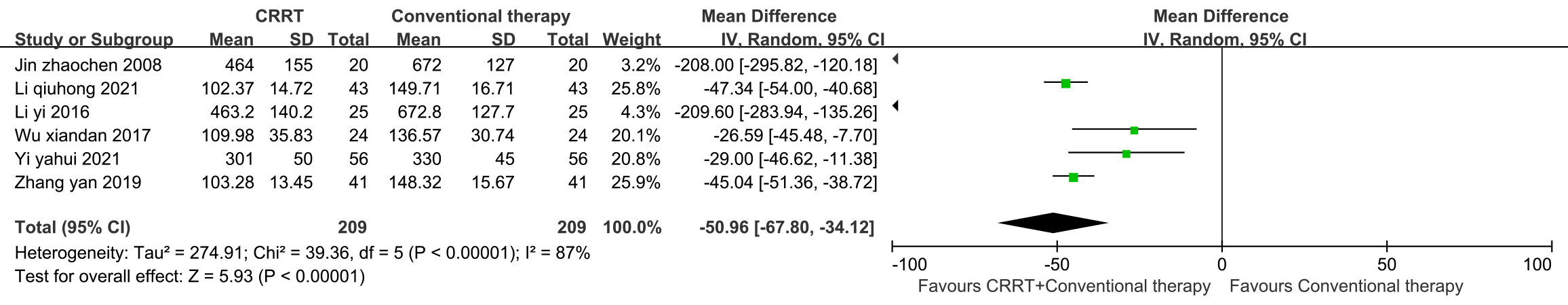

Supplement: Supplementary file 14 — Data S14 Forest‐plot of IL‐6 at 24 h. [file CRJ-19-e70045-s016.tif]

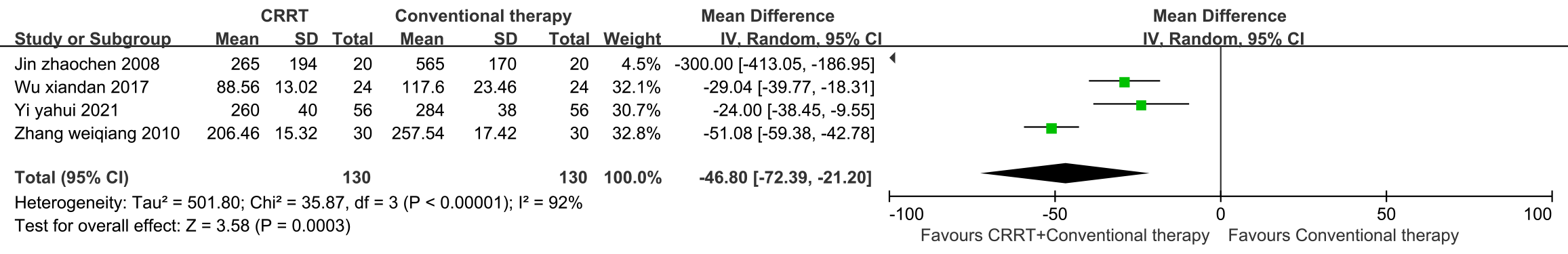

Supplement: Supplementary file 15 — Data S15 Forest‐plot of IL‐6 at 48 h. [file CRJ-19-e70045-s017.tif]

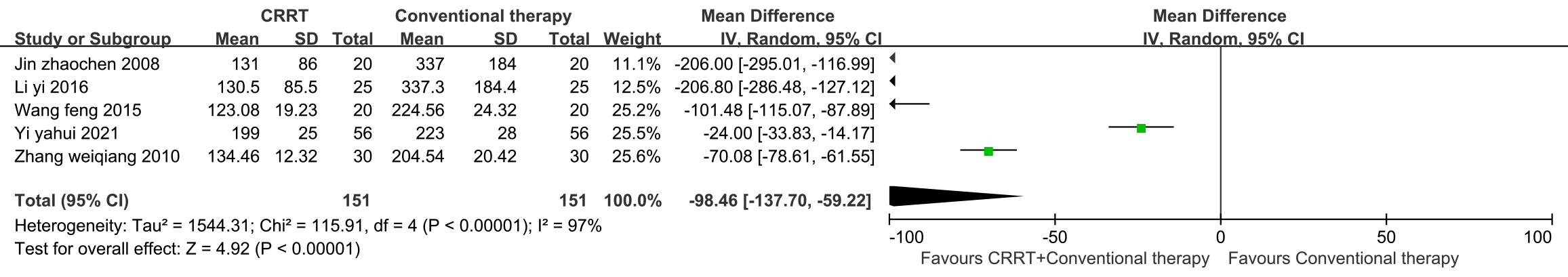

Supplement: Supplementary file 16 — Data S16 Forest‐plot of IL‐6 at 72 h. [file CRJ-19-e70045-s015.tif]

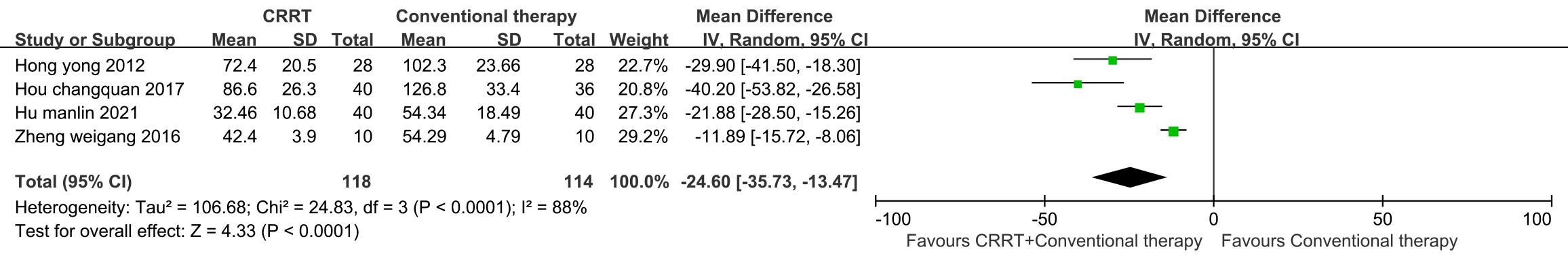

Supplement: Supplementary file 17 — Data S17 Forest‐plot of IL‐6 at 7 d. [file CRJ-19-e70045-s012.tif]
